# Solid/Gas Synthesis of Isobutyl Propionate Catalyzed by Packed-Bed CalB Cross-Linked Enzyme Aggregates (CLEA)

**DOI:** 10.1021/acsomega.5c09523

**Published:** 2025-11-24

**Authors:** Yahir Alejandro Cruz-Martínez, Carlos O. Castillo-Araiza, Edmundo Castillo-Rosales, Susana Velasco-Lozano, Sergio Huerta-Ochoa

**Affiliations:** 1 Department of Biotechnology, 27788 Universidad Autónoma Metropolitana-Iztapalapa, Av. San Rafael Atlixco 186, Col. Vicentina, Ciudad de México 09340, México; 2 Laboratory of Catalytic Reactor Engineering Applied to Chemical and Biological Systems, 27788 Universidad Autónoma Metropolitana-Iztapalapa, Ciudad de México 09340, México; 3 Department of Cellular Engineering and Biocatalysis. Instituto de Biotecnología, 119952 Universidad Nacional Autónoma de México, Av. Universidad 2001, Col. Chamilpa, Cuernavaca, Morelos 62210, México; 4 Instituto de Síntesis Química y Catálisis Homogénea (ISQCH), CSIC-Universidad de Zaragoza, C/Pedro Cerbuna, 12, Zaragoza 50009, Spain; 5 Aragonese Foundation for Research and Development (ARAID), Av. Ranillas 1-D, Zaragoza 50018, Spain

## Abstract

This research examines the viability and effectiveness of cross-linked enzyme aggregates (CLEA) of*Candida antarctica* lipase B (CalB)/bovine serum albumin (BSA) for the green synthesis of isobutyl propionate (isoPro) from propionic acid (aciP) and isobutyl alcohol (isoB). Solid/gas (S/G) biocatalysis is proposed as a more sustainable alternative to chemical synthesis, which involves toxic catalysts and harsh conditions, or plant extraction compounds, which are economically unfeasible for bulk production. A formulation containing 10 mg mL^–1^ BSA and 3% glutaraldehyde (GA) was selected based on CalB-CLEA’s demonstrated catalytic efficiency in *n*-heptane as solvent, along with its thermal and operational stability. Using 600 mg of the selected CalB-CLEA in the S/G bioreactor yielded 91.71% isoPro at a water activity (*a*_w_) of 0.52, with a nitrogen flow rate of 62 mL min^–1^ and a 1:4 acid/alcohol molar ratio, operating at 55 °C and 585 mmHg. Although 1.0 g of commercial CalB ImmoPlus achieved a higher yield (98.9%), its total turnover number (TTN) (mol product mol^–1^ enzyme) and specific space–time yield (STY_spe_) (g product L^–1^ h^–1^ mg^–1^ CalB) under steady-state conditions were 6- and 4-fold lower, respectively, than those of CalB-CLEA. Additionally, CalB-CLEA eliminates resin disposal costs, yielding similar outputs with lower enzyme loads while achieving comparable productivity. Ultimately, the S/G system incorporating CalB-CLEA outperforms both CalB ImmoPlus (in S/G) and CalB-CLEA (in *n*-heptane) in green, mass-based sustainability metrics, underscoring its strong potential for industrial-scale applications.

## Graphical abstract

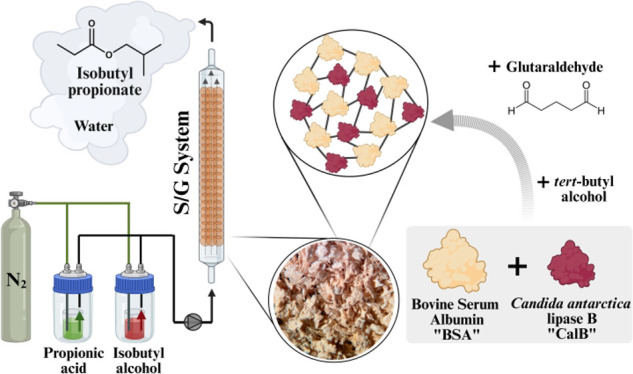

## 1 Introduction

Consumer demand tends toward natural products because the quality of the final product obtained by chemical synthesis can be affected by the toxic byproducts generated that are harmful to health.^1–3^ Natural volatile organic compounds (VOCs) have focused on human health because of their antioxidant, anti-inflammatory, anticancer, and antiobesity activities and their applications in the food, pharmaceutical, cosmetic, and personal care industries. Consumers consider natural flavors therapeutic, sustainable, and eco-friendly, so their demand in the global market is expected to grow on a large scale. The global flavor market was valued at US $5 billion in 2022 and is expected to grow at an annual growth rate of 6% from 2024 to 2032 (Aroma Chemicals Market Report By Source, Product, Application and Forecast, *Research and Markets*, March 2024). In recent years, there has been increasing interest in producing VOCs through biotechnological processes including *de novo* synthesis and biotransformation using enzymes or whole cells. This is attributed to the use of mild conditions, meaning that synthesis does not require toxic catalysts, and the resulted waste treatment is considered an environmentally safe solution due to its organic nature.^4^

The use of reaction media with organic solvents for synthesis reactions has been shown to be an advantageous approach to expanding the field of biocatalyst applications.^5^ In addition, ecological alternatives have been reported by using alternative solvents with low toxicity, low vapor pressure, good biodegradability, and easy recycling. Solvents that can fill the gap between volatile organic solvents and water are ionic liquids, deep eutectic solvents, supercritical fluids, and fluorinated solvents. Another nonconventional medium alternative for synthesizing organic compounds with enzymes was developed by Barzana et al.,^6^ called solid/gas (S/G) biocatalysis, involving solvent-free gas phase reagents. The process is carried out by immobilizing the biocatalyst on a porous solid support, through which the substrates flow in the gas phase through a carrier gas (N_2_), selectively converting them into specific products of commercial interest.^7^ Manipulating the operating conditions (temperature and pressure) in the S/G system allows for proper control over the thermodynamic activity of the water and substrate and the creation of a controlled microenvironment for the enzyme or cell. The biocatalyst is more thermostable in solid lyophilized form than in aqueous media or organic solvents.^8^ This allows the process to operate at elevated temperatures and for extended periods, increasing throughput and decreasing expenditure. On the other hand, low humidity decreases the risk of microbial contamination.^9,10^ The substrate directly interacts with the biocatalyst by not using solvents, eliminating the solvent’s toxicity and obtaining a high-purity natural product. Finally, the product can be recovered directly by condensing and reducing the separation costs. Therefore, this technology can be considered within White Biotechnology (Industrial Biotechnology), which uses enzymatic technology in synthesizing biobased chemicals and intensifying the process, developing sustainable and green solutions that reduce the carbon footprint.^11^ Although the S/G biocatalysis process already has several industrial applications that have demonstrated their technological feasibility,^7,12^ optimizing these systems involving biocatalysts remains a challenging task.^8^ Few studies are related to producing VOCs with applications in human health in the S/G system. Among the reactions studied are esterification,^13^ transesterification,^10^ alcoholysis,^14,15^ and enantioselective reduction.^16,17^ Leonard et al.,^18^ experimenting with an enantioselective acylation reaction catalyzed by lipase B of *Candida antarctica* (CalB), established that the S/G system is a useful tool for studying the influence of organic components on the enantioselectivity of lipases.

Biocatalysts are the core of the biocatalysis process, and the success of its development depends on carefully considering specific parameters such as catalytic activity, thermal and operational stability, reusability, productivity, and yield to ensure both effectiveness and practical applicability. Among these are the profitability and expansion potential involved in considering the cost of production and evaluating its viability for industrial applications as well as environmental and safety considerations that take into account relevant guidelines and standards. In addition, reuse should be considered by exploring methods to regenerate and reuse biocatalysts to improve the cost-effectiveness and reduce waste.

Therefore, the successful development of biocatalysts implies a comprehensive understanding of these parameters, among others. One of the main challenges associated with the development of biocatalysts is scaling, where the economic viability of the biocatalyst production cost must be considered. On a large scale, this cost can be prohibitive, affecting the economic viability of industrial applications. The change in scale can also be compromised due to limitations of transport phenomena (fluid dynamics and heat and mass transfer) that will be affected by the physicochemical characteristics of biocatalysts. Therefore, it is necessary to develop biocatalysts to produce natural aromatic compounds for human consumption that present efficient, more economical, and environmentally friendly processes.^11^

A key challenge in S/G biocatalysis systems is the development of immobilization strategies that ensure both catalytic efficiency and cost-effectiveness. Cross-linked enzyme aggregates (CLEA) provide a promising solution, as they allow for support-free immobilization using a cross-linking agent that permanently insolubilizes the enzyme. This strategy significantly reduces costs by avoiding expensive traditional resins, which require appropriate disposal after their useful life.^19^ Several CLEA-based approaches have been reported, including enzyme copolymers, cross-linked crystals, and protein-coated crystals. Notably, Sampaio et al.^20^ reviewed CLEA production for lipase-based biocatalysts, highlighting both challenges and opportunities in their preparation. CLEA technology offers significant advantages, such as enhanced enzyme stability and thermal resistance, high volumetric productivity and yields, and greater catalytic activity without the need for resins. Furthermore, it eliminates the requirement for highly purified enzymes, reducing costly purification steps, and represents a cost-effective process suitable for industrial applications. It also facilitates the easy recovery and recycling of the catalyst and allows the coimmobilization of multiple enzymes within a single aggregate, as seen in combi-CLEA. However, despite these benefits, CLEA preparation poses several challenges. The process requires careful analysis and selection of several factors, including the type and concentration of cross-linking agents, enzyme loading, and the presence of key amino acids for cross-linking, such as lysine. Moreover, CLEA often exhibit poor reproducibility and limited mechanical resistance. Controlling the size of the enzyme aggregates remains difficult, potentially leading to internal mass transfer limitations and reduced catalytic efficiency.

In response to the mass transfer limitations typically encountered in immobilized enzyme systems, CLEA-like biocatalysts offer a unique advantage: their tunable porosity enables enhanced substrate diffusion and product release, ultimately increasing the catalytic efficiency. Careful design of the CLEA structure balances stability and accessibility, making it a versatile biocatalyst for more sustainable and efficient biotransformations.

In this study, we exploit this feature to enable a greener and efficient synthesis of isobutyl propionate (isoPro) in an S/G bioreactor using CalB immobilized as CLEA with BSA as cofeeder protein (CalB-CLEA). Key parameters were evaluated to maximize the biocatalyst’s activity, stability, and recyclability. To the best of our knowledge, this is the first report to apply CLEA technology for the clean and efficient synthesis of VOCs in an S/G system, marking a novel and pioneering contribution to the field of biocatalysis.

## 2 Materials and Methods

### 2.1 Chemicals

Propionic acid (aciP), isobutyl alcohol (isoB), isobutyl propionate (isoPro), and glutaraldehyde 50% (GA) were obtained from either Sigma Chemical Co. or Aldrich (St. Louis, MO, USA). Methanol was acquired from J.T. Baker (Mexico). All these chemicals had a high purity level (>98%); substrate and product purity was confirmed by gas chromatography analysis before being used in experimental procedures. Bovine serum albumin (BSA) was acquired from Fisher Scientific (Spain). Finally, we gratefully acknowledge Novozymes (Denmark) for generously donating the CalB Lipozyme enzyme.

### 2.2 Production of Cross-Linked Enzyme Aggregates (CLEA) from CalB

The section aimed to assess how the use of bovine serum albumin (BSA) and the concentration of cross-linking agent glutaraldehyde (GA) affect the body (particle size) of CLEA. It is essential to increase the size of the particles for their use in the S/G system and pack them inside the S/G bioreactor while ensuring that the enzyme’s catalytic activity is not compromised. Five concentrations of BSA (5, 10, 15, 20, and 40 mg mL^–1^) and three concentrations of GA (0.5, 1, and 3% v/v) were used to produce the CLEA. First, the required weight of solid BSA in 2 mL Eppendorf tubes was placed. Prior to immobilization, commercial CalB Lipozyme was buffer-exchanged into 50 mM sodium phosphate buffer at pH 7.0 using a tangential flow filtration system (Amicon Ultra-15) equipped with a 10 kDa molecular weight cut off membrane. This procedure effectively removed formulation excipients such as glycerol, sorbitol, sodium benzoate, and potassium sorbate while retaining intrinsic trace macromolecules present in the product (minor amounts of nucleic acids and negligible levels of 21.5 and 66 kDa proteins).^21^ The resulting enzymatic extract exhibited a protein concentration of 3 mg mL^–1^, as determined by the Bradford assay, corresponding to an activity of 3.96 U mL^–1^. Subsequently, 70 μL of the CalB solution (enzyme load: 7% v/v) and the appropriate volume of 50 mM sodium phosphate buffer (pH 7.0) were added to reach 10% of the total reaction volume, considering the inclusion of glutaraldehyde (GA) within this same proportion. The BSA was completely dissolved using a Mini Spin. Next, 900 μL of *tert*-butanol (precipitant) was added and vigorously shaken for 30 s, and the required volume of GA was added and vigorously shaken for another 30 s. The mixture was subsequently placed in a rotary shaker (Biosan “Multi Bio RS-24”) at 400 rpm and incubated for 3.5 h at room temperature, shaking every 30 s at a 60° angle. After cross-linking, the mixture was centrifuged at 12,000 rpm and 25 °C for 10 min to remove the supernatant and unreacted GA. The CLEA (pellet) was washed three times (900 μL × three times) using 50 mM sodium phosphate buffer (pH 7). After each wash, the sample was centrifuged at 12,000 rpm and 4 °C, and the supernatants were collected to quantify the enzyme released during washing. The hydrolytic and synthetic activities of the resulting CalB-CLEA were assessed to evaluate the effectiveness of the immobilization process. Subsequently, samples displaying the best characteristics, in terms of synthesis capacity and thermal stability, were selected. The impact of water activity (*a*_w_) on synthesis and the number of catalytic cycles were then evaluated to assess potential activity loss and determine biocatalyst stability, as detailed below. To perform the S/G synthesis of isoPro, CalB-CLEA was lyophilized to obtain a freeze-dried biocatalyst.

### 2.3 Hydrolytic Activity and Thermal Stability of the Biocatalyst

The immobilization process was followed by measuring the hydrolytic activity of both soluble and immobilized CalB using a colorimetric assay with *p*-nitrophenyl butyrate (pNPB) as the substrate. The reaction mixture was composed of 0.5 mM pNPB in 50 mM sodium phosphate buffer (pH 7) with 1% acetonitrile (used to prepare the concentrated stock solution of 50 mM pNPB). For the assay, 10 μL of soluble or immobilized CalB-CLEA suspension was placed in a 96-well microplate followed by 200 μL of the reaction mixture. To ensure accurate and reproducible dosing, the CalB-CLEA suspension was kept under continuous gentle mixing prior to and during pipetting to maintain homogeneity of the particle distribution. Aliquots of 10 μL were dispensed using pipet tips with trimmed ends to minimize clogging and variability. Each experimental condition was assayed in at least five technical replicates, and data points with a coefficient of variation (CV) > 20% were excluded from analysis. The plate was then incubated at 30 °C with continuous shaking. To quantify the formed *p*-nitrophenol, absorbance was recorded at 348 nm using a BioTek Epoch2 microplate reader for at least 5 min.

The CalB-CLEA activity (U mL^–1^) was calculated using the following equation:

$$A=\frac{\Delta AV_{T}}{\varepsilon lV_{e}}$$

where *A* is the CalB-CLEA activity (U mL^–1^), Δ*A* is the slope (min^–1^), *V*_T_ is the total reaction volume (mL), *V*_e_ is the enzyme volume (mL), ε is the molar extinction coefficient (ε for *p*-nitrophenol = 5.4 mM^–1^ cm^–1^), and *l* is the path length, where 360 μL corresponds to 1 cm. One unit (U) was defined as the amount of biocatalyst required to produce 1 μmol *p*-nitrophenol per minute. After the activity (U mL^–1^) was determined, the specific activity of the biocatalyst was calculated by dividing U mL^–1^ by the mass (mg) of CalB present in the CLEA excluding BSA.

For the thermal stability tests, 150 μL of CalB-CLEA suspension was resuspended in 250 μL of 50 mM sodium phosphate buffer (pH 7) and incubated at 55 °C for 2 h in a thermoblock. Subsequently, the residual enzymatic activity was measured by using the aforementioned method.

### 2.4 Synthetic Activity of CalB-CLEA in *n*-Heptane

The CalB-CLEA obtained using the procedure described in Subsection [Sec sec2.2] were equilibrated at water activity (*a*_w_) of 0.11 by incubating them at 4 °C in a LiCl-saturated water solution for 48 h inside a sealed container. The CLEA were placed separately within the container, ensuring exposure to the controlled humidity generated by the LiCl solution without direct contact with the liquid phase. The synthesis activity of CalB-CLEA was assessed by measuring isoPro production using the CalB-CLEA content in 200 μL of CalB-CLEA suspension after removing water by organic solvent washes. The reaction medium consisted of 1 mL of *n*-heptane containing 400 mM isoB and 100 mM aciP (molar ratio 4:1). The reaction was conducted at 55 °C and 180 rpm for 3 h. Subsequently, the concentration of the produced ester (isoPro) was determined by using gas chromatography (GC). The synthetic activity of the enzyme preparation was expressed as the total micromoles of isoPro produced per milligram of immobilized enzyme (μmol mg^–1^), along with the product yield relative to the limiting reagent (aciP).

### 2.5 Effect of *a*_w_ on Ester Synthesis

To investigate the impact of water activity (*a*_w_) on ester synthesis, 5 mg of previously freeze-dried CalB-CLEA was weighed and allowed to equilibrate for 48 h at 4 °C with saturated solutions of salts with known *a*_w_ values: LiCl (0.11), MgCl_2_ (0.32), Mg­(NO_3_)_2_ (0.52), and NaCl (0.75). Afterward, 1 mL of 400 mM isobutanol and 100 mM propionic acid (molar ratio 4:1) in *n*-heptane were added. The mixture was then independently kept at 55 °C and 180 rpm for 30, 90, 180, and 270 min. Duplicate samples were taken at each time, and after the specified reaction time, they were centrifuged at 12,000 rpm and 4 °C for 10 min. The supernatant was then filtered through a nylon filter (0.45 μm) and analyzed by GC.

### 2.6 Recyclability of CalB-CLEA

To assess the recyclability, 5 mg of freeze-dried CalB-CLEA was used and allowed to equilibrate with the saturated solution chosen in the previous stage (*a*_w_ = 0.52) for 48 h at 4 °C. Subsequently, 1 mL of the same reaction medium (400 mM isobutanol and 100 mM propionic acid in *n*-heptane) was added and incubated at 55 °C for 2 h and 180 rpm. Afterward, the mixture was centrifuged, and the supernatant was filtered and analyzed by GC. The formed CalB-CLEA pellet was washed twice with *n*-heptane (1 mL each wash) with centrifugation steps in between. Finally, 1 mL of a new reaction medium was added, and the process was repeated until 10 reaction cycles were completed.

### 2.7 Synthesis of isoPro in the S/G Bioreactor

The S/G bioreactor employed in this study was homemade (Figure 1). The essential components of the S/G system described previously^22^ encompassed:

i. An insulated acrylic chamber equipped with convection-based heaters, designed to maintain precise thermal conditions at 55 °C. Temperature control was achieved using a Vernier system and an Arduino-based program for real-time monitoring at multiple points inside the chamber (two near the bioreactor and two near the substrate containers).
ii. The system was configured so that a carrier gas (ultrahigh-purity nitrogen, N_2_ 99.999%, Linde) passed through containers holding the liquid-phase reactants, allowing the gas stream to become saturated with vaporized substrates before entering the packed-bed reactor. The vaporization rates of propionic acid (aciP) and isobutanol (isoB) were controlled by maintaining constant temperature, pressure (585 mmHg, corresponding to the atmospheric pressure in Mexico City), and N_2_ flow rate. Prior to connecting the packed column, the system was stabilized for 2 h under steady-state conditions using an empty column.
iii. Rotameters were used to precisely regulate N_2_ flow at the column inlet, ensuring stable bubbling and vapor generation. The combination of isothermal operation, controlled carrier gas flow, and substrate temperature regulation maintained the desired 1:4 molar ratio of aciP to isoB in the gas phase throughout the reaction.
iv. To operate the reactor, the reaction mixture was supplied by feeding the substrates in the gas phase at a constant total nitrogen flow rate (*Q*_N_2__) of 62 mL min^–1^, resulting in molar flow rates of *ṅ*_isoB_ = 27.72 and *ṅ*_aciP_ = 7.23 μmol min^–1^. The bioreactor employed in this study consisted of a glass column (22 cm length × 0.8 cm internal diameter), with the biocatalyst held in place by cotton plugs positioned below and above the bed.
v. A collection system was implemented at the column outlet to facilitate the storage of different species within microtubes. Each microtube contained 0.5 mL of methanol and was maintained at −5 °C using a recirculating chiller. Sampling was performed in accordance with the experiment’s timing requirements to ensure data accuracy and reliability.

**Figure 1:**
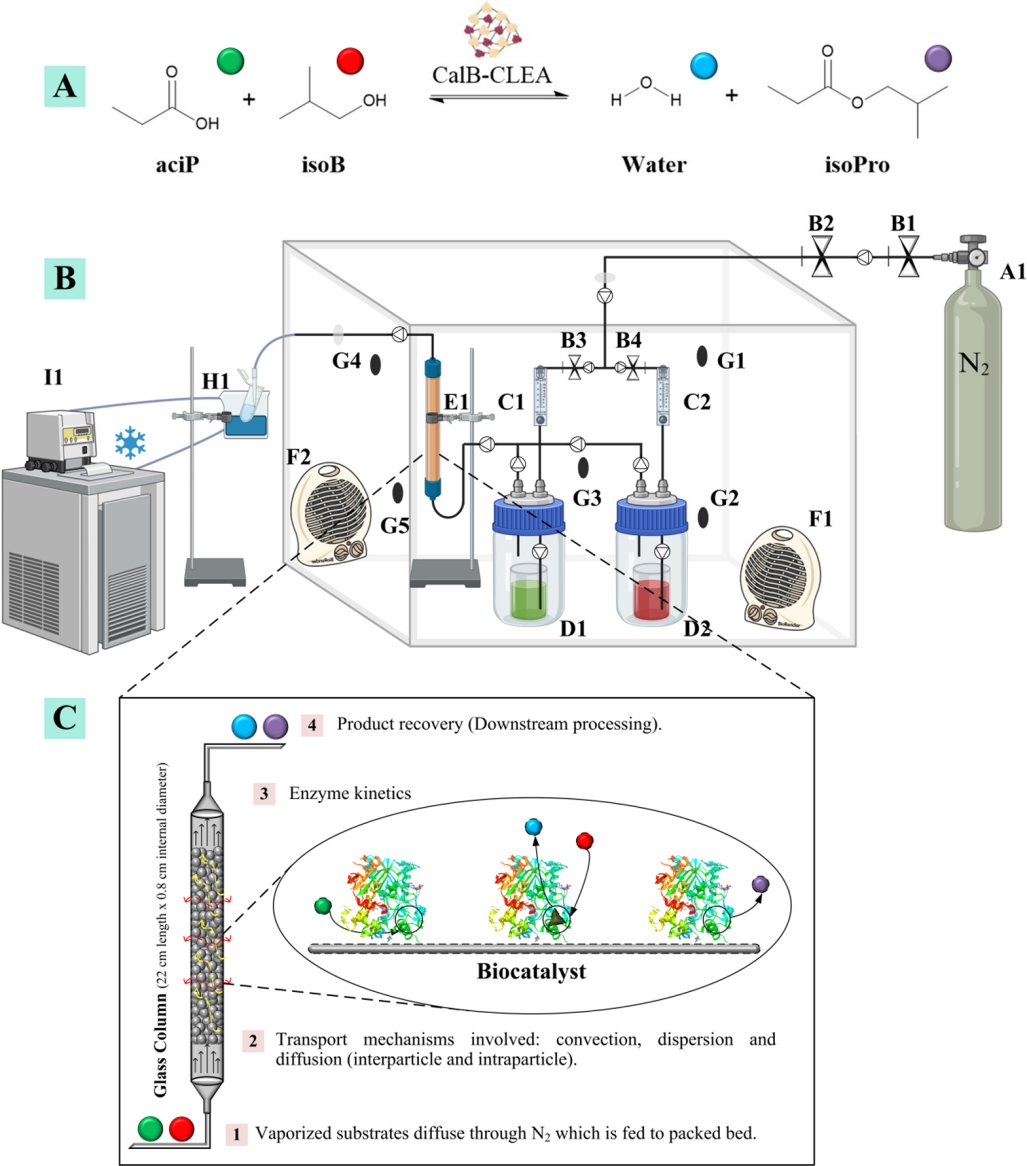
(A) Substrates and products involved in the esterification reaction using the proposed biocatalyst. (B) General components of the S/G system. A1: gas carrier tank, B1–B4: valves, C1–C2: rotameters, D1–D2: reactants containers, E1: packed bed column, F1–F2: convection heaters, G1–G5: thermocouples, H1: condenser (methanol at −5 °C), and I1: recirculating chiller. (C) Bioreactor process stages.

### 2.8 Water Activity and Biocatalyst Load Effect on the S/G System

To establish the initial *a*_w_, the same saturated saline solutions applied in organic media analysis were used [LiCl (0.11), MgCl_2_ (0.32), Mg­(NO_3_)_2_ (0.52), and NaCl (0.75)]. A 200 mg of freeze-dried CalB-CLEA was brought to equilibrium with these solutions for 72 h at 4 °C (*a*_w_ for all samples was also measured using an Aqualab CX-2 instrument) and subsequently packed into the column. This biocatalyst loading corresponds to a bed height of approximately 5.2 cm, a bed volume of 2.61 cm^3^, and a packing density of 0.077 g cm^–3^.

For biocatalyst load assessment, 100, 200, 400, and 600 mg of CalB-CLEA, previously equilibrated at *a*_w_ = 0.52, were packed into the S/G bioreactor, resulting in bed heights of approximately 2.5, 5.2, 8.5, and 14.6 cm, respectively. These bed heights correspond to volumes of 1.25, 2.61, 4.27, and 7.33 cm^3^, corresponding to an average packing density of 0.081 ± 0.005 g cm^–3^. All experiments were conducted at 55 °C, with samples collected every 10 min over 180 min.

### 2.9 Stability, Recyclability, and Comparison of CalB-CLEA in the S/G system

To assess the stability and recyclability in the S/G system, 600 mg of freeze-dried CalB-CLEA was equilibrated with the selected saturated solution (*a*_w_ = 0.52) for 72 h at 4 °C and subsequently were packed into the column. A total *Q*_N_2__ = 62 mL min^–1^ was used, corresponding to molar flows of *ṅ*_isoB_ = 36.52 and *ṅ*_aciP_ = 9.17 μmol min^–1^. The reaction rates of isoPro production were monitored continuously for 9 h. After each run, the packed column was disconnected from the substrate flow and stored at 4 °C, and the mixture was equilibrated again with the same saturated solution. The next day, the column was reconnected, and the kinetic assay was repeated for another 9 h. This cycle was repeated daily until the biocatalyst lost 50% of its activity. All experiments were performed at 55 °C and 585 mmHg, with samples taken every 20 min.

Finally, for the evaluation of commercial CalB ImmoPlus, 1 g of biocatalyst, previously equilibrated at *a*_w_ = 0.11 (as selected in a prior study),^22^ was packed into the same glass column, resulting in a bed height of approximately 11.5 cm and a packed-bed volume comparable to that of CalB-CLEA. This loading corresponded to a bed volume of 5.78 cm^3^ and a packing density of 0.173 g cm^–3^. The rates of isoPro production were monitored continuously over 9 h, with samples collected every 20 min.

### 2.10 Gas Chromatography Assays

Synthesis samples were analyzed by using an Agilent 7820 A gas chromatograph equipped with a flame ionization detector. To facilitate separation, a DB-HEAVYWAX column with specific dimensions (60 m in length, 0.250 mm in internal diameter, and a 25 μm film thickness) was employed, and N_2_ gas served as the carrier medium throughout the chromatographic process. The temperature protocol applied during the analysis comprised an initial phase with an isothermal hold at 40 °C for a duration of 5 min; subsequently, the temperature increased at a rate of 25 °C/min until reaching 90 °C; and finally, there was a further ramp with a rate of 30 °C/min, ultimately achieving a temperature of 200 °C. This temperature was maintained for a period of 2 min. Both the detector and injector were consistently maintained at a temperature of 200 °C, and each sample was introduced into the system using an injection volume of 1 μL. Retention times were as follows: 4.65 min for methanol, 7.13 min for isoPro, 7.29 min for isoB, and 10.85 min for aciP. Quantification was performed using external standard calibration curves for each compound (see Figures S1 and S2), ensuring accurate and reproducible measurements across all samples.

### 2.11 Scanning Electron Microscopy (SEM)

SEM images were obtained with the assistance of the UAM-Iztapalapa scanning electron microscopy laboratory by using a Jeol 7600F SEM and a gold coating. Particle size distributions and SEM analysis parameters were determined with the ImageJ software.

## 3 Results and Discussion

### 3.1 Hydrolytic Activity and Thermostability

CalB-CLEA was produced using *tert*-butanol as a precipitating agent and glutaraldehyde (GA) as a cross-linker, as this solvent had been successfully employed for this purpose in previous studies.^23,24^ Due to its hydrophobicity and bulky, branched structure, *tert*-butanol exerts a comparatively mild effect on the enzyme’s native conformation. It facilitates rapid precipitation while promoting the formation of compact, homogeneous aggregates (Figure 5), preserving enzymatic activity through interactions with the hydrophobic region near the CalB active site.^25^ The resulting aggregates are stabilized by noncovalent interactions with the precipitant. Conversely, Díaz-Vidal et al. (2019)^24^ reported that precipitants such as acetone, ethanol, and acetonitrile result in poor catalytic activity retention, likely due to conformational alterations of the enzyme. In contrast, the primary goal of incorporating BSA was to enhance both the body mass and the porosity of CalB-CLEA while maintaining enzymatic activity, ensuring their applicability in an S/G biocatalytic system. To achieve this, we evaluated the effect of the concentrations of BSA and GA on the increase in body mass and the catalytic performance of the final CLEA biocatalyst. Given the BSA solubility of 40 mg mL^–1^ in water, we evaluated concentrations of 5, 10, 20, and 40 mg mL^–1^, which were cross-linked with varying GA concentrations (0.5, 1.0, and 3.0%). This resulted in a CalB-CLEA biocatalyst dubbed as CalB-CLEA-B_*x*_G_*y*_, where *x* represents the BSA concentration and *y* corresponds to the GA concentration. For example, CalB-CLEA-B_5_G_0.5_ refers to the biocatalyst prepared with 5 mg mL^–1^ of BSA and 0.5% of GA. Under the screening cross-linking conditions, all CalB-CLEA achieved immobilization yields exceeding 95% (Table S1). Moreover, the increases in the body mass of CalB-CLEA and the intensity of the orange color (due to the GA reaction) were directly proportional to the increases in BSA and GA concentrations, respectively, achieving the most robust immobilization with CalB-CLEA-B_40_ at various levels of GA concentration (Figure S3).

Figure 2A and Table S1 show the hydrolytic activity and thermal stability of CalB-CLEA prepared with varied concentrations of BSA and GA. A negative correlation is observed between protein loading (body mass increase) and hydrolytic activity, as shown on the left *Y* axis. The highest activity (20.09 ± 0.60 U mg^–1^) was reached with CalB-CLEA-B_5_G_1_, which used the lowest BSA concentration. In contrast, CalB-CLEA-B_40_G_3_, prepared with the highest BSA concentration, exhibited the lowest activity, approximately 6 times lower than the maximum observed. Conversely, increasing the GA concentration from 0.5 to 1% during CalB immobilization enhanced the hydrolytic activity. However, further increases beyond 1% GA led to a decline in activity across all treatments, except for the CalB-CLEA prepared with 40 mg mL^–1^ BSA, where activity consistently decreased with rising GA concentration. Guauque Torres et al. (2014)^26^ investigated the synthesis of CalB-CLEA using BSA as a cofeeder protein and glutaraldehyde (GA) as a cross-linker, evaluating the effect of the GA-to-protein mass ratio on CLEA activity. They found that increasing the GA concentration enhanced the recovered catalytic activity, with the highest value observed at a ratio of 1.67 mg of GA per mg CalB (equivalent to approximately 120 mg of GA, estimated, not reported). These results align closely with those obtained in this work. Notably, our work extends this understanding by demonstrating that even higher GA concentrations (up to ∼200 mg of GA per mg of CalB) can be used without compromising catalytic activity, offering valuable insights into the upper limits of GA usage in CLEA preparation.

**Figure 2:**
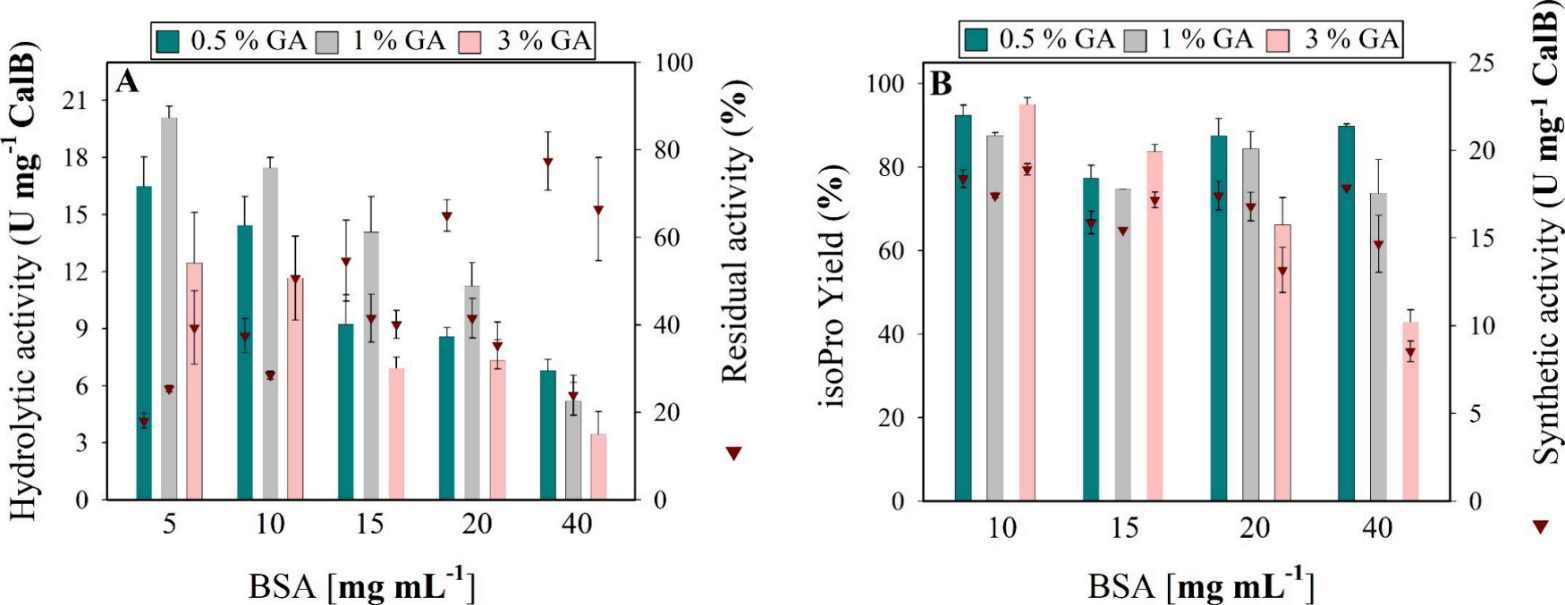
(A) Effect of BSA and GA concentrations on the hydrolytic activity (bars) and thermal stability after 2 h of incubation at 55 °C (inverted triangles) of CalB-CLEA. (B) Synthetic activity of CalB-CLEA in *n*-heptane (400 mM isoB and 100 mM aciP) for 3 h at 55 °C and 180 rpm at different BSA and GA concentrations. The results represent the average of three independent replicates, with error bars indicating the standard deviation from the mean.

The increase in body mass on CalB-CLEA (directly proportional to the BSA concentration) enhances thermal stability, which was measured as the percentage of activity retained after 2 h of incubation at 55 °C (Figure 2A, right-side *Y* axis). These results are aligned with those previously reported, where the effect of BSA has a positive impact on CLEA’s thermal stability.^26,27^ This can be attributed to the protective layer formed by the cross-linked BSA, which enhances enzyme stability, incorporating more reactive functional groups for the GA reaction (the Lys amine group essentially). The most thermostable biocatalyst was CalB-CLEA-B_40_G_0.5_, maintaining 77.4 ± 6.7% of residual activity, while the least stable was CalB-CLEA-B_5_G_0.5_, retaining only 18.1 ± 1.7%. Additionally, increasing the GA concentration has a positive effect on thermostability but only up to biocatalysts containing 10 mg mL^–1^ BSA. Beyond this threshold, the trend reversed, with a decrease in the retained activity as the GA concentration increased from 0.5 to 3%. This decrease may be attributed to excessive cross-linking with the BSA, which could disrupt the intramolecular interactions essential for maintaining the enzyme’s three-dimensional structure. This structural destabilization likely leads to reduced enzymatic activity, a phenomenon previously observed in similar studies.^28^

The observed trends in Figure 2A served as selection criteria, allowing us to evaluate the impact of the body mass increase and cross-linking extent in determining the optimal preparation conditions. Although CalB-CLEA prepared with the lowest BSA concentration (5 mg mL^–1^) showed the highest hydrolytic activity, these biocatalysts were discarded due to their low thermal stability and insufficient CLEA body mass, both of which are critical factors for S/G synthesis conditions. In contrast, while CalB-CLEA prepared with 10 mg mL^–1^ BSA showed lower activity, their significantly higher thermal stability and twice the body mass were key determinants for their selection. The ability of BSA to form condensates in crowded environments increased both the density of the condensates and the mean radius as the BSA concentration rose.^29^ Moreover, we also consider the synthetic activity in the organic solvent of the obtained CalB-CLEA since the target enzymatic activity needed to be evaluated for its application in the S/G biocatalysis system.

### 3.2 Synthetic Activity in Organic Solvent

The different CalB-CLEA prepared in the previous section were evaluated for isoPro synthesis in *n*-heptane, enabling us to assess the impact of BSA and GA concentrations on the catalytic performance of the biocatalysts in synthesis reactions. As shown in Figure 2B, after 3 h of reaction at 55 °C, biocatalysts prepared with BSA concentrations of 10 and 15 mg mL^–1^ exhibited a positive correlation between increased GA concentration and isoPro synthesis yield. Among them, CalB-CLEA-B_10_G_3_ achieved the highest product yield, reaching 95.0 ± 1.62%. Subsequently, a negative effect of increasing the GA concentration during cross-linking was observed as the BSA content in CLEA increased. The lowest product yields were obtained with biocatalysts prepared with 3% GA, with the most pronounced decrease at 40 mg mL^–1^ BSA, which exhibited the lowest product yield (42.9 ± 2.93%). Notably, although CalB-CLEA-B_10_G_3_ achieved the highest isoPro yield, 7 out of the 12 different CalB-CLEA biocatalysts resulted in isoPro yields exceeding 80%. This highlights the remarkable synthesis capacity and thermal stability of the produced CalB-CLEA, emphasizing the effect of the variables evaluated.

Across all synthesis tests, the data indicate that both GA and BSA concentrations significantly influence the synthesis performance of the CalB-CLEA. Specifically, at lower BSA concentrations, a higher GA concentration is required to enhance the stability of the biocatalyst. Conversely, as BSA concentration increases, biocatalyst stability decreases due to a higher extent of cross-linking caused by the higher BSA protein content (a 4-fold increase from 40 to 10 mg mL^–1^). This excessive cross-linking may restrict enzyme flexibility and mobility, ultimately impairing the catalytic efficiency. These results are consistent with those reported by Cui et al.,^30^ who demonstrated that bovine pancreatic lipase-based CLEA prepared with BSA as cofeeder exhibited enhanced activity, reaching a maximum at 0.05 mg mL^–1^ BSA. At higher BSA concentrations, however, activity decreased to levels even lower than those of CLEA prepared without BSA. In addition, they reported that glutaraldehyde concentrations above 1% (v/v) led to excessive cross-linking, reducing active-site accessibility and thereby diminishing the catalytic activity of lipase-BSA CLEA.

Regarding synthetic activity, we reported the achieved isoPro yield in *n*-heptane at 55 °C over a 2 h reaction period. To calculate the synthetic specific activity of CalB-CLEA, the total molar mass of the produced isoPro was divided by the mass of the employed biocatalyst, considering only the enzyme mass and excluding the mass of BSA. The results are expressed in synthetic activity units (U), defined as μmol of isoPro per minute and per milligram of immobilized CalB in the CLEA form. The effect of BSA and GA concentrations on CLEA synthetic activity in *n*-heptane followed a trend similar to that observed for hydrolytic activity with pNPB as substrate (Figure 2B, right *Y* axis). The most synthetically active biocatalyst, CalB-CLEA-B_10_G_3_, achieved 18.91 ± 0.32 U mg^–1^, while the least active, CalB-CLEA-B_40_G_3_, exhibited a 2.22-fold lower specific activity. The increased GA concentration has a more pronounced detrimental effect on CalB-CLEA at higher BSA concentrations, reducing the specific activity from 17.88 ± 0.11 to 8.54 ± 0.58 U mg^–1^ (B_40_G_0.5_ to B_40_G_3,_ respectively). It is crucial to highlight this information, as future optimizations may explore the possibility of increasing the enzyme concentration during the preparation of immobilized biocatalysts while preserving the structural integrity achieved in this study. This approach could enhance the enzymatic activity, ultimately improving the productivity per mass of CalB-CLEA.

Finally, Table S1 summarizes the results for all prepared CalB-CLEA biocatalysts. D, E, and L (highlighted in bold) were identified as the most suitable candidates for potential application in the S/G biocatalysis system. Their selection was based on their synthetic activity in organic solvents, thermal stability, and the ease of quantifying their enzymatic hydrolytic activity as an initial screening criterion for future trials. These correspond to CalB-CLEA-B_10_G_3_, CalB-CLEA-B_20_G_0.5_, and CalB-CLEA-B_40_G_0.5_, which were selected in further studies.

### 3.3 Effect of Water Activity (*a*_w_) on the IsoPro Synthesis in *n*-Heptane

Once the best biocatalysts were selected, we investigated the effect of *a*_w_ on the isoPro synthesis in *n*-heptane over 4.5 h of reaction kinetics. An increase in *a*_w_ positively influenced the synthesis rate of CalB-CLEA (Figure 3). This finding contrasts sharply with previous reports by various authors,^22,31,32^ which reported that lowering *a*_w_ enhances synthesis rates in lipase-catalyzed reactions, specifically in esterification. A decrease in *a*_w_ impacts the reaction thermodynamics by shifting the equilibrium toward product synthesis due to the reduced water content.^33^ Additionally, it is closely linked to the enzyme conformation mobility and flexibility, which are critical for substrate interaction. Lipases typically require minimal water content to maintain their activity, and increased rigidity has been associated with improved synthetic performance.^7,8^

**Figure 3:**
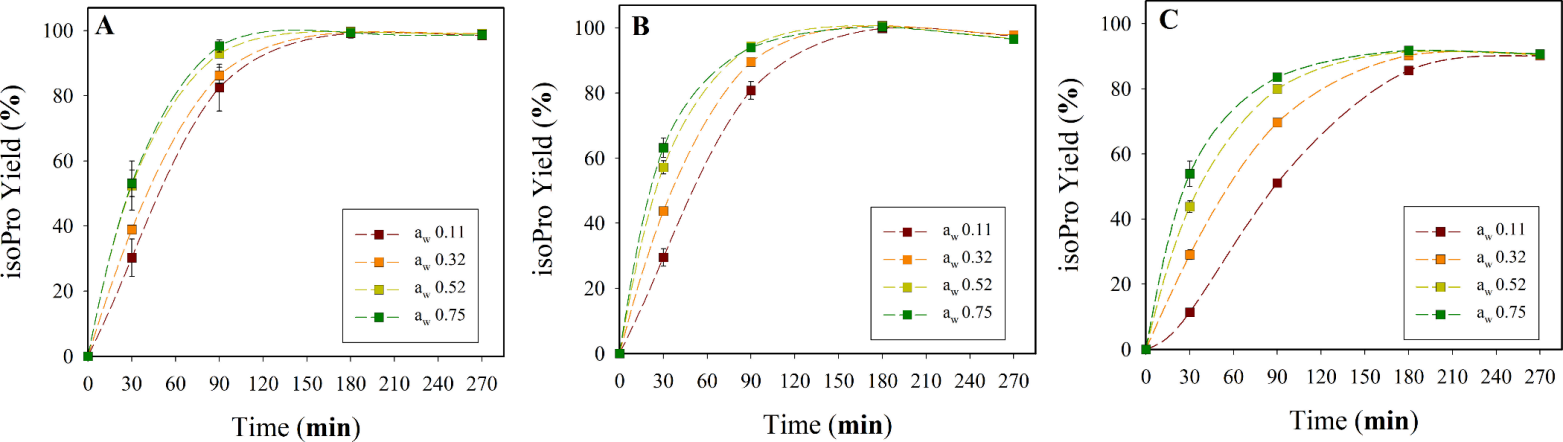
Effect of *a*_w_ on isoPro synthesis by 5 mg of CalB-CLEA (400 mM isoB and 100 mM aciP in *n*-heptane) for 270 min at 55 °C and 180 rpm. (A) CalB-CLEA-B_10_G_3_, (B) CalB-CLEA-B_20_G_0.5_, and (C) CalB-CLEA-B_40_G_0.5_. The results represent the average of three independent replicates, with error bars indicating the standard deviation from the mean.

After 250 min, most biocatalysts reached comparable isoPro yields (98–100%), except for CLEA-B_40_G_0.5_, which plateaued at ∼90%. At shorter reaction times (<90 min), differences in initial rates reflected both *a*_w_ levels and CLEA formulation. CalB-CLEA-B_20_G_0.5_ at *a*_w_ = 0.75 showed the highest rate (3.30 ± 0.15 μmol min^–1^; Figure 3A) followed by CalB-CLEA-B_20_G_0.5_ (*a*_w_ = 0.52) and CalB-CLEA-B_10_G_3_ (*a*_w_ = 0.75 and 0.52), which displayed rates of 2.92 ± 0.15, 2.70 ± 0.19, and 2.60 ± 0.20 μmol min^–1^, respectively (Figure 3B). Elevated BSA concentrations within CLEA (40 mg mL^–1^) markedly lowered activity: CalB-CLEA-B_40_G_0.5_ at *a*_w_ = 0.11 achieved only 0.74 ± 0.21 μmol min^–1^ (Figure 3C). Increasing BSA content also widened the performance gap between high and low *a*_w_, and rates at *a*_w_ = 0.75 were 1.74-, 2.22-, and 3.60-fold higher than those at *a*_w_ = 0.11 for CalB-CLEA-B_10_G_3_, -B_20_G_0.5_, and -B_40_G_0.5_, respectively. These results suggest that high BSA loadings increase CLEA density and rigidity, reducing internal porosity and slowing substrate diffusion. Under low *a*_w_, this effect is exacerbated by insufficient enzyme hydration, which further limits conformational flexibility and active-site accessibility.^27^ Together, these factors account for the observed reduction in catalytic efficiency and increase in the initial rate ratio across different water activities among the evaluated biocatalysts. In this context, the presence of BSA in CalB-CLEA likely contributes to a “water-reservoir” effect since its hydrophilic domains can retain bound water and influence the local hydration state of the enzyme. Under excessive BSA loading (e.g., 40 mg mL^–1^) and low glutaraldehyde concentration (0.5%), unreacted hydrophilic groups may remain exposed, enhancing water adsorption and thereby altering enzyme microenvironment and catalytic performance.

Overall, *a*_w_ modulates reaction kinetics primarily during the initial phase, whereas extended reaction times minimize these differences, leading to similar final yields. Nevertheless, excessive BSA incorporation decreases the overall product yield by ∼10%, likely due to diffusion limitations and reduced effective catalytic turnover.

Although no significant differences were observed in the kinetic behavior at *a*_w_ values of 0.75 and 0.52, in most cases, *a*_w_ = 0.52 was selected for subsequent experiments (biocatalyst comparative on Figure S4). From a thermodynamic perspective, this choice ensures the lowest possible water activity, favoring the reaction equilibrium toward product formation in further studies. This consideration is particularly relevant since the tests conducted so far were performed in batch systems without removing the water produced during esterification. However, this limitation may not be as critical in continuous systems such as the S/G system.

Afterward, we evaluated the operational stability of the CalB-CLEA biocatalysts through repeated batch reaction cycles for isoPro synthesis in *n*-heptane. All three tested biocatalysts demonstrated outstanding operational stability, retaining over 80% of their catalytic activity after 10 reaction cycles, each lasting 2 h at 55 °C and 180 rpm, totaling 20 h of reaction time (Figure 4).

**Figure 4:**
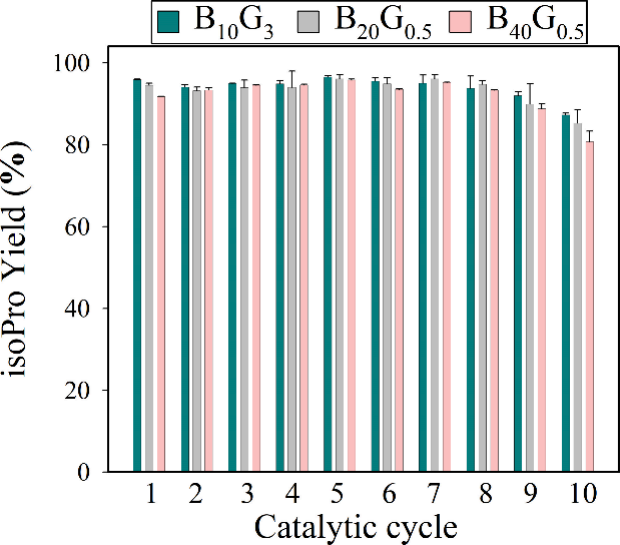
Operational stability of CalB-CLEA during repeated 2 h isoPro synthesis cycles. The results represent the average of three independent replicates, with error bars indicating the standard deviation from the mean.

For practical applications and in alignment with the objectives of this work, CalB-CLEA-B_10_G_3_ was selected as the most suitable biocatalyst for its application in the S/G system. SEM and stereoscopy imaging was employed to examine its internal morphology. The biocatalyst forms enzyme aggregates ranging from approximately 0.77 to 2.30 μm, with an average area of 1.64 ± 0.75 μm^2^ and an average diameter of 1.41 ± 0.34 μm. The calculated polydispersity index (PDI) of 0.058 [PDI= (standard deviation/mean aggregate diameter)^2^] indicates low polydispersity, approaching monodispersity. Furthermore, all aggregates were clearly identified as being fully enveloped by BSA (Figure S5 and Figure 5A). The size and shape of these aggregates have been previously reported by Shoevaart et al.,^23^ albeit without the presence or absence of BSA. The presence of BSA appears to contribute to the formation of slightly more homogeneous aggregates. The observed black voids might indicate free spaces created when some CalB aggregates are released and lost during multiple washing steps. Conversely, Figure 5B illustrates the morphology of the CalB-CLEA, revealing folded sheet-like structures of varying sizes, estimated to range from approximately 10 to 100 μm. Saikia et al.^34^ previously reported particle sizes of around 50 μm using a lower BSA concentration than that applied in this study. Lastly, Figure 5C presents the freeze-dried CalB-CLEA in a macromolecular form, observed under a stereoscope, where the presence of sheets and their cross-linking at multiple points are noticeable. Finally, the theoretical (geometric) specific surface area of the biocatalyst was estimated based on the aforementioned mean aggregate diameter and the bulk density (0.081 g cm^–3^), yielding a value of 52.5 ± 5.6 m^2^ g^–1^.

**Figure 5:**
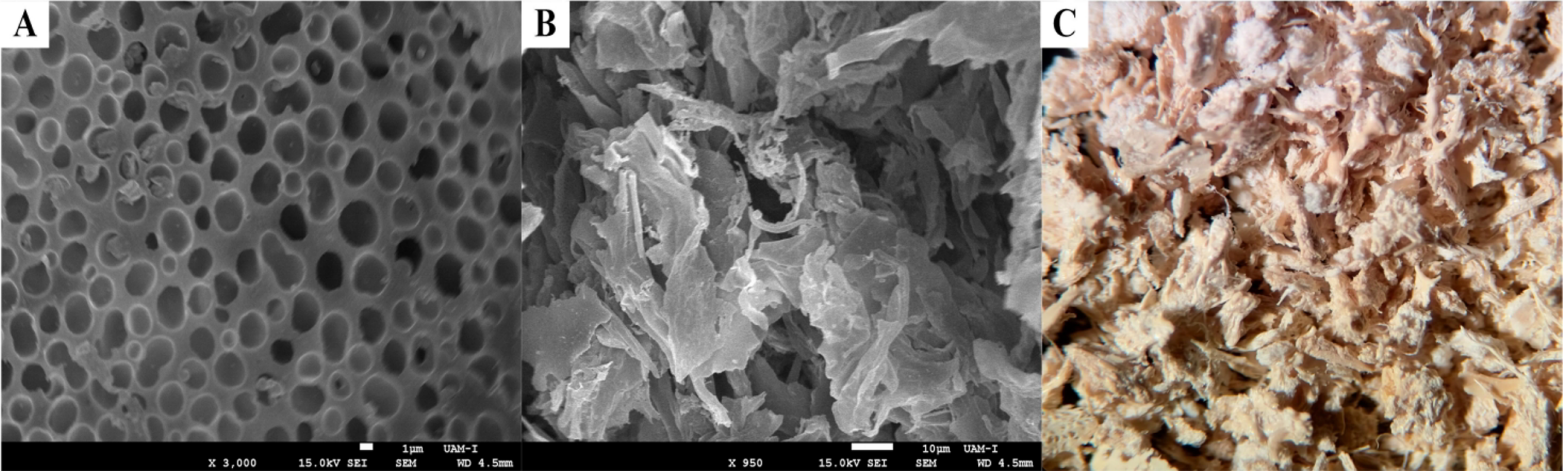
SEM images from CalB-CLEA: (A) 1 μm scale with ×3000 magnification, (B) 10 μm scale with ×950 magnification, and (C) macromolecular image from a stereoscope.

### 3.4 Solid/Gas Synthesis of IsoPro Catalyzed by CalB-CLEA

To continue our study, we evaluated the effect of *a*_w_ on isoPro synthesis in an S/G system. To this aim, we packed 200 mg of freeze-dried CalB-CLEA-B_10_G_3_ previously equilibrated at the specified *a*_w_ inside the glass column reactor. The results showed that increasing *a*_w_ positively influenced the isoPro yield, reaching its highest average yield of 48.68 ± 1.06% at *a*_w_ = 0.52 during the steady state, which was achieved after approximately 120 min (Figure 6A). However, beyond this *a*_w_ value, the catalytic activity of the biocatalysts in the S/G system decreased, with the lowest average isoPro yield of 31.13 ± 1.40% at *a*_w_ = 0.75, where the steady state was reached in around 90 min.

**Figure 6:**
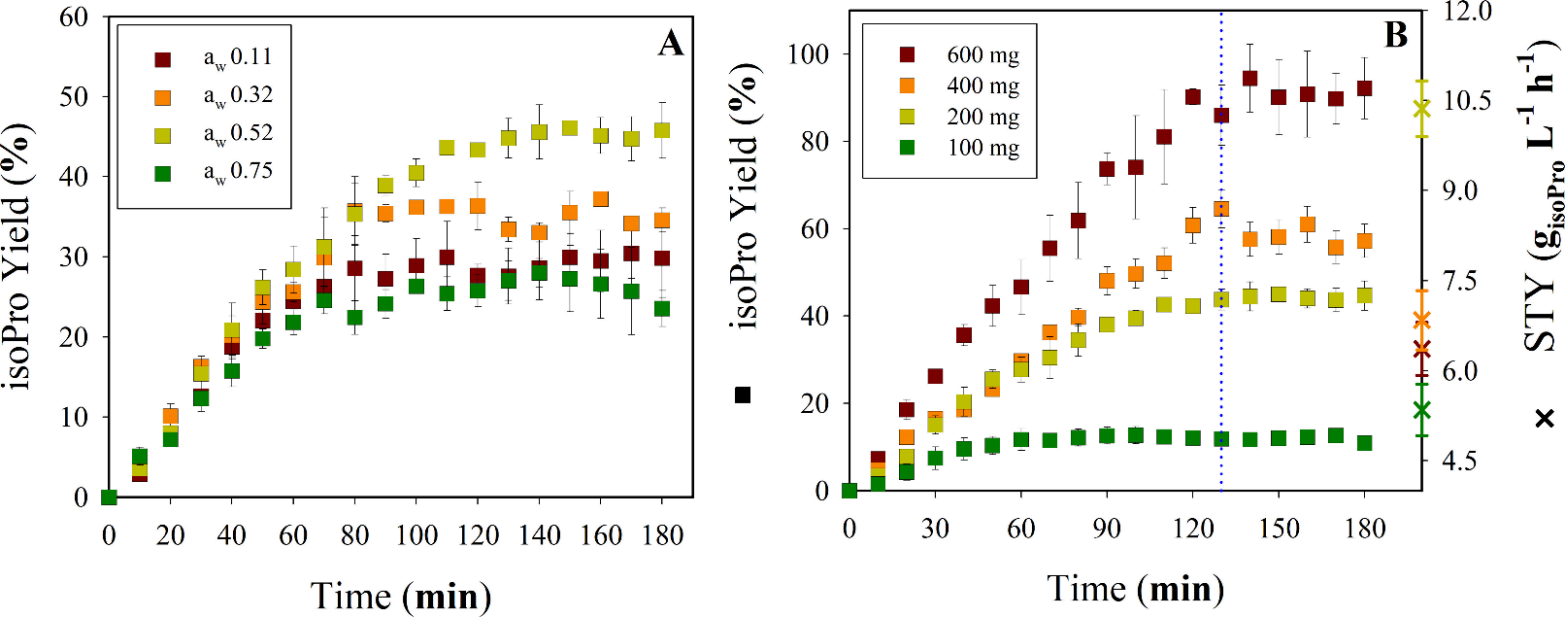
(A) Effect of *a*_w_ on isoPro synthesis in an S/G system packing 200 mg of freeze-dried CalB-CLEA-B_10_G_3_. (B) Effect of the biocatalyst load in the S/G packed-bed bioreactor. STY: space–time yield; average value during steady state (start at the blue dotted line). S/G system conditions: nitrogen flow rate (*Q*_N_2__) of 62 mL min^–1^, with average molar fluxes of 7.23 ± 0.40 and 27.72 ± 1.41 μmol min^–1^ for aciP and isoB, respectively (molar ratio ∼1:4), at 55 °C and 585 mmHg. The results represent the average of three independent replicates, with error bars representing the standard deviation from the mean.

This result contrasts with observations in the organic phase at *a*_w_ = 0.52 and 0.75, where the highest product formation rates, faster steady-state achievement, and yields approaching 100% were obtained. In the S/G system, however, a decline in catalytic activity at *a*_w_ ≥ 0.75 is attributed to the formation of transient liquid films by capillary condensation of water.^35^ These films may be intermittently removed by the gas flow, creating microdomains where esterification and hydrolysis compete,^36^ leading to reduced conversions similar to those at *a*_w_ = 0.11. This effect is absent in *n*-heptane due to its low dielectric constant, which limits water mobility and stabilizes the thin layer of bound water around the enzyme, preserving its active conformation. Collectively, these findings underscore the critical influence of *a*_w_ on enzyme performance and its system-dependent behavior. Based on these observations, *a*_w_ = 0.52 was selected as the optimal condition for subsequent S/G experiments.

After evaluating the effect of the water activity in the S/G bioreactor, we analyzed the packed biocatalyst mass to maximize the isoPro yield, aiming for values closer to 100%. Increasing the biocatalyst load to 600 mg resulted in the highest product yield (89.32 ± 4.08%), whereas a 200 mg load achieved only 43.81 ± 0.954% (Figure 6B, left axis). Notably, doubling or halving this amount of biocatalyst did not lead to a proportional increase or decrease in reaction rate or product yield; for example, using 100 mg of CalB-CLEA resulted in an isoPro production yield of only 12.06 ± 0.529%, significantly lower than half of what was observed with 200 mg at steady state. Similar findings were reported by Csanádi et al. (2012)^13^ using an S/G bioreactor for ethyl acetate production with immobilized CalB as biocatalyst. Tripling the initial enzyme load increased the product yield 4-fold, but doubling it led to only a minimal improvement. On the other hand, this was also reflected in the space–time yield (STY), which exhibits its maximum at 200 mg of biocatalyst (10.25 ± 0.441 g isoPro L^–1^ h^–1^) (Figure 6B, right axis). However, as denoted above, at this reduced mass, the system achieved only ∼40% isoPro yield at the fixed substrate feeding rate.

The difference observed in the performance of the biocatalyst was primarily influenced by the residence time in the packed bed and the adsorption–reaction–desorption cycle necessary for the enzyme to conduct the reaction. When too little CalB-CLEA is packed, the residence time appears to be insufficient for efficient conversion. Increasing bed height improves isoPro yield by extending residence time, as a higher catalyst amount naturally enhances substrate interaction. However, this effect seems to plateau at a critical point (not determined in this study), beyond which the adsorption–reaction–desorption time becomes the dominant factor in reaction kinetics. This may explain the minimal difference in conversion between 200 and 400 mg. Based on the results of this work, the adsorption–reaction–desorption process exhibits a characteristic time that cannot be bypassed even when doubling the catalyst amount. While this does not hinder an increase in conversion efficiency, it does impose limitations on analyzing the initial reaction rate. This characteristic time is affected not only by the enzyme’s catalytic activity but also by the high proportion of BSA in the formulated catalyst. Due to its intrinsic properties, BSA can interact with organic compounds through hydrophilic interactions and hydrogen bonding, potentially impacting substrates isoB and aciP, as well as the product isoPro. These interactions may slow the reaction and delay product release from the effluent. This behavior is further supported by the observation that tripling the catalyst load to 600 mg enhances product formation until a threshold is reached, after which the accumulated product is released more quickly. Our group previously reported a similar phenomenon using the same bioreactor but with CalB ImmoPlus as the biocatalyst.^22^ In that study, various phenomena, including adsorption, desorption, and adsorption–reaction–desorption, were analyzed in isoPro production. A distinct characteristic time associated with these processes was identified, significantly influencing the early stages of the reaction kinetics.

Additionally, we compared the performance of our CalB-CLEA biocatalyst with that of the commercial CalB ImmoPlus for isoPro synthesis in the S/G bioreactor. To this end, we plotted progress curves of isoPro production under the optimal experimental conditions identified (Figure 7A and Figures S6 and S7). The right *Y* axis shows isoPro production relative to the immobilized enzyme load, expressed as the accumulated total turnover number (TTN), calculated as the moles of isoPro produced per mole of enzyme. According to Wunschik et al.,^37^ the commercial CalB ImmoPlus contains approximately 10% (w/w) enzyme. In our experiments, this corresponds to an enzyme load of around 100 mg of enzyme per gram of biocatalyst. In contrast, the CalB-CLEA formulation contains only ∼21 mg of enzyme per gram of biocatalyst. Remarkably, despite the lower enzyme content, CalB-CLEA achieved comparable product yields, highlighting the superior catalytic efficiency of the cross-linking approach. Notably, the CalB-CLEA system delivered 6 times more accumulated moles of isoPro per mole of enzyme within the same reaction time (10 h) while maintaining an average space–time yield (STY) only 1.5 times lower than that of the commercial system. Specifically, CalB-CLEA reached 12.13 ± 0.15 × 10^3^ mol isoPro mol^–1^ enzyme and 8.185 ± 0.28 g isoPro L^–1^ h^–1^, while CalB ImmoPlus reached 2.30 ± 0.09 × 10^3^ mol isoPro mol^–1^ enzyme and 11.79 ± 0.40 g isoPro L^–1^ h^–1^. An important aspect to consider is the specific volumetric productivity parameter (STY_spe_) (g of isoPro L^–1^ h^–1^ mg^–1^ of CalB). When normalized to enzyme mass, CalB-CLEA exhibited a value 4.4 times higher than that of CalB ImmoPlus (0.65 ± 0.02 and 0.15 ± 0.01, respectively). This normalization provides a more accurate measure of intrinsic catalytic efficiency and clearly demonstrates the superior performance of the CalB-CLEA biocatalyst developed in this study.

**Figure 7:**
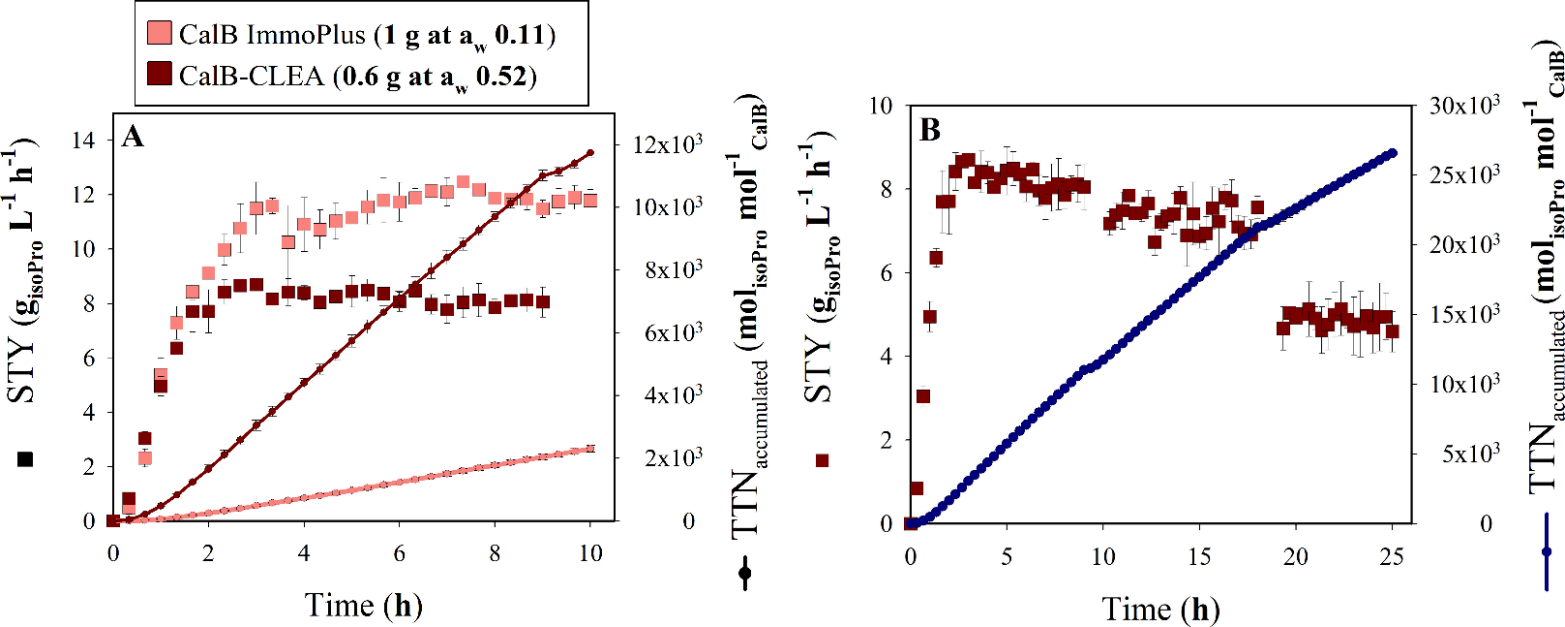
(A) Comparison of space–time yield (STY) and total turnover number (TTN) for isoPro synthesis using CalB ImmoPlus and CalB-CLEA, produced under 10 h kinetics at their respective optimal *a*_w_ conditions using the S/G system. (B) Flow reactor stability and activity metrics for isoPro synthesis under 25 h using 600 mg CalB-CLEA at *a*_w_ = 0.52 in the S/G system. Data represent the mean of three independent replicates, with error bars indicating the standard deviation.

Additionally, unlike CalB ImmoPlus, where the enzymatic activity is primarily confined to the particle surface,^38^ CalB-CLEA displays a more homogeneous enzyme distribution throughout the particle matrix, facilitating improved substrate accessibility and more efficient utilization of active sites. Furthermore, the hydrophobic nature of the ECR1030M support used in CalB ImmoPlus may further restrict substrate diffusion due to steric hindrance and/or specific interactions such as hydrogen bonding.^22^

Finally, we evaluated the stability and recyclability of the immobilized biocatalyst in the S/G system. The formulated biocatalyst exhibited high stability, maintaining its highest isoPro yield during 9 h under the specified temperature and pressure conditions. Notably, 7 h of this operation occurred in a steady state, resulting in the maximum isoPro production with an average conversion of 91.02 ± 3.16%. After this working period, we had to shut down the reactor as our gas-supply system is not automated for overnight operation. To do so, the glass column containing the packed biocatalyst was removed from the flow system and stored at 4 °C until the following day, without unpacking the catalyst. On the following day, after reconnecting the packed column to the substrate’s flow, the reactor reached a steady-state isoPro yield of 81.35 ± 3.63%, indicating a slight decrease in synthetic capacity (<10%) after a total of 18 h of operation. On the third day, an average conversion of 54.01 ± 1.97% was achieved due to a decline in catalytic capacity, approaching the value representing 50% of the initial catalytic activity (Figure S8). As a result, it was decided to terminate the kinetics after 25 h of operation. IsoPro production could be significantly enhanced by optimizing the design of the S/G system to allow continuous operation, thereby preventing interruptions in the kinetic process, which in our case had a detrimental effect on biocatalyst activity. It is suggested that maintaining the biocatalyst in contact with adsorbed substrates, followed by storage, reduces its catalytic performance upon reactivation.

Despite these operational limitations, the results obtained are promising. The STY during the assessed kinetic period averaged 8.11 ± 0.18 g of isoPro L^–1^ h^–1^ during the steady state on the first day (Figure 7B). This value declined to 7.33 ± 0.33 g of isoPro L^–1^ h^–1^ on the second day and then further dropped to 4.88 ± 0.16 g of isoPro L^–1^ h^–1^ by the final day. By adjusting these data to the Arrhenius deactivation model, we estimated an approximate biocatalyst’s half-life of 25.86 h, accumulating a TTN value of 26.580 ± 0.015 × 10^3^ mol of isoPro mol^–1^ CalB. The natural logarithm of this value yielded 10.18, which is close to the lower limit proposed for a highly stable heterogeneous catalysts (Ln TTN > 11.25) according to the two-dimensional map of metrics defining the productivity and operational stability of immobilized enzymes in continuous operations reported by Bolivar and Lopez-Gallego.^39^ It is noteworthy that, using the proposed metrics map, the STY natural logarithm value obtained was 2.11, indicating low activity due to its position below the proposed lower limit (Ln STY > 4.7). While the initial metrics may seem somewhat negative at first glance, it is imperative to consider factors that were not previously addressed, such as the nature of the compound, its product price, and its market size. These elements serve as crucial benchmarks and offer valuable insights.

Considering this significant subject, Meissner and Woodley^40^ reported ranges for typical metric values expected for economically feasible biocatalytic processes. The market price of isobutyl propionate is approximately $110–155 kg^–1^ (Sigma-Aldrich, TCI America), making it a high-priced or high-value product (>$ 100 kg^–1^). Taking this into account, we determined that the metrics obtained with our biocatalyst in the S/G system fell within the described optimal ranges: rate or STY (mass product/reaction volume/reaction time) of 1–10 g L^–1^ h^–1^ (8.11 ± 0.18 g isoPro L^–1^ h^–1^), yield (mass product/mass substrate) > 90 (91.2 ± 3.16%), and specific yield (mass product/mass enzyme) of 50–500 g g^–1^ protein (104.871 ± 0.06 g isoPro g^–1^ CalB from TTN reported previously). Overall, CalB-CLEA in the S/G system represents a promising alternative for a more sustainable and economically feasible production of natural esters in industry such as isoPro.

### 3.5 Green and Sustainability Metrics

Due to the excellent performance of CalB-CLEA in synthesizing isoPro, the sustainability of the CalB-CLEA biocatalyst was assessed and compared with the performance of the commercial CalB ImmoPlus. Mass-based green metrics (Table S2) were calculated for three processes: CalB-CLEA (5 mg in *n*-heptane and 600 mg in the S/G system) and CalB ImmoPlus (1 g in the S/G system).

The evaluated green metrics included reaction mass efficiency (RME), mass productivity (MP), and carbon economy (CE).^41,42^ RME and MP were defined as ratios of product mass to the total mass of reactants alone and to the total mass including biocatalysts and solvents, respectively. CE was defined as the ratio of the total carbon mass in the product to the total carbon mass in the reactants. Additionally, the atom economy (AE) and stoichiometric factor (SF) were calculated for the compared systems to evaluate their overall efficiency. The calculated green metrics serve as benchmarks for evaluating the synthesis efficiency, with an ideal process represented by a value of 1 for each parameter. Both the CalB-CLEA biocatalyst (600 mg) and CalB ImmoPlus (1 g) demonstrated the best overall performance, although the lowest values were consistently observed for RME and MP % (Figure 8A). This reduction is primarily due to the 1:4 molar ratio used in the synthesis, which requires an excess of isoB to achieve optimal isoPro yields.

**Figure 8:**
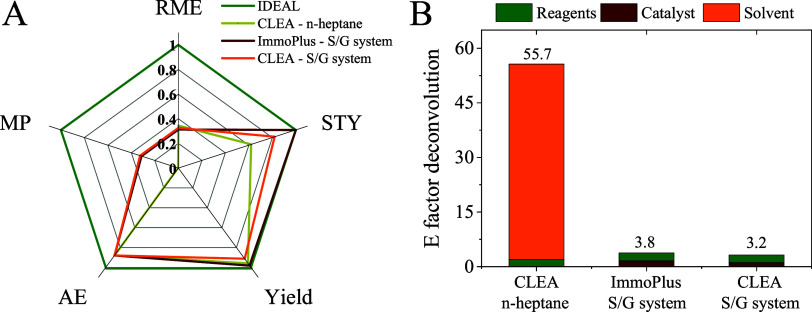
Green sustainability metrics for isoPro synthesis. (A) AE: atom economy (%) = (mol wt of product/sum of mol of reactants). Yield = (mol of product isoPro/mol of limiting reactant aciP). RME: reaction mass efficiency = (mass of product/total mass of reactants). MP: mass productivity = (mass product/total mass including solvents). STY = g_isoPro_ L^–1^ h^–1^ (ideal STY corresponds to 10 g L^–1^ h^–1^, which is in the upper range of STY for high-priced products). (B) *E* factor = total mass of waste/mass of final product. Employed biocatalyst mass: 5 mg of CLEA in *n*-heptane, 1 g of ImmoPlus in the S/G system, and 600 mg of CLEA in the S/G system.

Calculating the *E* factor (kg waste/kg product)^41,42^ and mass productivity (MP) for each process requires special consideration due to the unconventional nature of the S/G system, which operates without solvents and uses nitrogen as an inert carrier gas. To better reflect its environmental impact, calculations were made with and without including nitrogen, as it is debatable to classify atmospheric nitrogen (78.08% of air) as a conventional solvent or waste like *n*-heptane. The CalB-CLEA in the S/G system achieved an *E* factor of 3.22, approximately 17 times lower than that of the same biocatalyst in *n*-heptane (55.7), demonstrating its superior sustainability for isoPro synthesis. Moreover, product recovery is straightforward, as it coexists only with unreacted substrates, enabling high purity through fractional condensation. Additionally, water does not impact the *E* factor in this setup, further enhancing the system’s green profile. While CalB ImmoPlus showed a lower *E* factor than CalB-CLEA in *n*-heptane (3.8 vs 55.7), it was still 1.2 times higher than that of CalB-CLEA in the S/G system.

Ultimately, Figure 8B illustrates the comparative effect of each reaction component on *E* factor deconvolution. The highest value, 55.7, was obtained with CalB-CLEA in *n*-heptane, with a large contribution of the solvent used in the reaction, unlike the S/G system, where this will not happen. Furthermore, the smallest value, 3.2, was recorded with CalB-CLEA, followed by 3.8 with CalB ImmoPlus. In both cases, the characteristic reagent size area is similar; however, the catalyst area for CalB ImmoPlus is larger. While this could be seen as a disadvantage due to the greater amount of catalysts used during the tests, it is crucial to highlight what these catalyst areas represent in both cases. It is well-known that 90% of the commercial product CalB ImmoPlus mass comes from the synthetic polymeric support. In contrast, CalB-CLEA consists almost entirely of protein, making it more biodegradable and maybe compostable. This offers clear advantages concerning waste reduction (and a more manageable waste stream), operational sustainability, and overall process performance.

Finally, we benchmarked our CalB-CLEA-B_10_G_3_ biocatalyst in the S/G system against previously reported strategies for isoPro synthesis in nonconventional media (Table S2). Varma and Madras^43^ employed supercritical CO_2_ with CalB Novozym 435 (analogous to CalB ImmoPlus) at 50 °C and 100 bar, achieving 95% conversion after 2.5 h. Despite comparable conversion, their STY was slightly lower than ours (7.1 vs 8.2 g L^–1^ h^–1^), while their process generated substantially more waste, with a 14-fold higher *E* factor (45.5 vs 3.2) and 40× greater total waste (67.6 g vs 1.7 g). Additionally, the high-pressure operation introduces significant energy demands and process complexity, limiting scalability. Their system also achieved a 1.2-fold lower TTN than ours (20,921 mol isoPro mol^–1^ CalB vs 26,580 mol isoPro mol^–1^ CalB).

Kuperkar et al.^44^ reported a solvent-free approach using CalB Novozym 435, reaching 92.5% conversion after 5 h at 40 °C and 200 rpm. Although their system achieved a 5.9-fold higher STY (48.4 vs 8.2 g L^–1^ h^–1^), this was accomplished by using isoB extensively as both substrate and solvent, leading to comparable mass productivity (32.7 vs 31.3%) but 15.7-fold more waste (26.7 g vs 1.7 g). Additionally, working with a liquid mixture rich in isoB introduces added complexity in downstream purification, potentially increasing costs and energy requirements. While their TTN was 3.8-fold higher than that of our CalB-CLEA system, it is important to note that our S/G setup is not optimized for fully continuous, long-term operation; the packed column must be disconnected every 9 h, stored at 4 °C, and subsequently reconnected, which can negatively impact the biocatalyst’s stability and performance.

Overall, these comparisons highlight that CalB-CLEA-B_10_G_3_ in the S/G system combines competitive conversion (91.2%) with superior environmental performance, substantially reducing total waste and the *E* factor compared with other media. Notably, if the mass of the biobased, biodegradable cofeeder (BSA) used to prepare the CLEA is excluded, the *E* factor further decreases from 3.2 to 2.1, reinforcing the advantage of this approach as a greener and more scalable route for isoPro synthesis.

## 4 Conclusions

This work underscores the potential of cross-linked lipase aggregates (CalB-CLEA) as a promising biocatalyst for greener ester synthesis in S/G biocatalysis. Our results demonstrate that CalB-CLEA exhibit significant hydrolytic activity, enhanced thermal and operational stability, and excellent synthetic catalytic performance. Furthermore, our findings indicate that isoPro synthesis in organic solvents (using *n*-heptane) was significantly improved by increasing GA concentration, leading to a competitive isoPro yield. Additionally, the increase in the biocatalyst body due to the presence of BSA in CalB-CLEA facilitated the formation of a porous structure, improving its catalytic performance in the S/G system. This modification enabled the CLEA biocatalyst to achieve a competitive conversion percentage of product yields in operation times comparable to those obtained with commercial CalB ImmoPlus under similar operating conditions. Therefore, CalB-CLEA emerges as a highly effective and more sustainable biocatalyst for ester synthesis in the S/G system. Its eco-friendly nature, cost-effectiveness, and ability to replace expensive traditional resins position it as a superior alternative for industrial applications in biocatalysis.

## Supplementary Material



## Notes

Supporting tables including a summary of the results from different treatments applied during CalB-CLEA production and green and sustainability mass metrics and supporting figures including substrates and product standard gas chromatography curves, the effect of BSA concentration on the CLEA body mass, effect of water activity (*a*_w_) on isoPro synthesis by CalB-CLEA, particle size distribution histogram, chromatograms acquired throughout the isoPro production kinetics in the S/G system, and the kinetics of isoPro synthesis in the S/G system (PDF)

## Abbreviations

CalB: *Candida antarctica* lipase B
BSA: bovine serum albumin
GA: glutaraldehyde
CLEA: cross-linked enzyme aggregate
S/G: solid/gas system
isoPro: isobutyl propionate
aciP: propionic acid
isoB: isobutyl alcohol
*a*_w_: water activity
TTN: total turnover number
STY: space–time yield
STY_spe_: specific space–time yield
CalB-CLEA-C_*x*_G_*y*_: treatment *x*: mg mL^–1^ BSA, *y*: % GA
AE: atom economy
RME: reaction mass efficiency
MP: mass productivity
CE: carbon economy
