# Supplementary material for: Solid/Gas Synthesis of Isobutyl Propionate Catalyzed by Packed-Bed CalB Cross-Linked Enzyme Aggregates (CLEA)
Source: ACS Omega. 2025 Nov 24;10(48):59692–705. doi: 10.1021/acsomega.5c09523 (PMC12771433; doi:10.1021/acsomega.5c09523)
Supplement: Supplementary file 1 [file ao5c09523_si_001.pdf]

# Supporting Information

## Solid/Gas Synthesis of Isobutyl Propionate Catalyzed by Packed-Bed CalB Cross-Linked Enzyme Aggregates (CLEA)

Yahir Alejandro Cruz-Martínez[1], Carlos O. Castillo-Araiza[2], Edmundo Castillo-Rosales[3], Susana Velasco-Lozano[4,5*], Sergio Huerta-Ochoa[1*]

[1] Department of Biotechnology, Universidad Autónoma Metropolitana-Iztapalapa, Av. San Rafael Atlixco 186, Col. Vicentina, 09340, Ciudad de México, México.

[2] Laboratory of Catalytic Reactor Engineering Applied to Chemical and Biological Systems, Universidad Autónoma Metropolitana-Iztapalapa, Ciudad de México, México.

[3] Department of Cellular Engineering and Biocatalysis. Instituto de Biotecnología. Universidad Nacional Autónoma de México, Av. Universidad 2001, Col. Chamilpa, 62210 Cuernavaca, Morelos, México

[4] Instituto de Síntesis Química y Catálisis Homogénea (ISQCH), CSIC-Universidad de Zaragoza, C/ Pedro Cerbuna, 12, 50009 Zaragoza, Spain.

[5] Aragonese Foundation for Research and Development (ARAID), Av. Ranillas 1-D, 50018, Zaragoza, Spain.

[*] Corresponding authors:

sho@xanum.uam.mx (SHO)

svelasco@unizar.es (SVL)

# Table of contents


Supporting Tables ........................................................................ S3

**Table S1.** Results summary from treatments of CalB-CLEA production ................. S3

**Table S2.** Green and sustainability mass metrics summary...................................... S4

Supporting Figures ........................................................................ S5

**Figure S1.** GC-FID calibration curves...................................................................... S5

**Figure S2.** GC-FID analysis of commercial standards ............................................. S6

**Figure S3.** Effect BSA concentration (mg $mL^{-1}$) in CLEA body mass using 3% GA. ........................................................................................................................ S7

**Figure S4.** Comparison of isoPro synthesis by CalB-CLEA´s at $a_w$: 0.52 ................ S8

**Figure S5.** Particle size distribution of CalB-CLEA-$B_{10}G_3$ determined at a 1 µm scale and 3000 magnification SEM using ImageJ software. .............................................. S9

**Figure S6**. GC-FID analysis of the substrate feed introduced into the S/G bioreactor ........................................................................................................................ S10

**Figure S7.** GC–FID monitoring of isoPro formation kinetics in a packed-bed CalB–CLEA reactor operated under the S/G system.......................................................... S11

**Figure S8**. isoPro synthesis kinetics in the S/G system. .......................................... S12

Supporting References ........................................................................ S13

## Supporting Tables

**Table S1.** Results summary from treatments of CalB-CLEA production using different concentrations of BSA and GA.

| CLEA | BSA (mg mL$^{-1}$) | GA (% v v$^{-1}$) | Ψ (%) [a] | Recovered specific activity (%) [b] | Thermal stability (%) [c] | isoPro yield (%) [d] |
|---|---|---|---|---|---|---|
| A | 5 | 0.5 | 99.7 | 89.5 ± 8.3 | 18.1 ± 1.7 | NE |
| B | 10 | 0.5 | 99.3 | 78.3 ± 8.2 | 37.5 ± 3.9 | 92.3 ± 2.5 |
| C | 15 | 0.5 | 99.6 | 53.0 ± 9.0 | 54.7 ± 9.2 | 79.7 ± 3.3 |
| **D** | **20** | **0.5** | **99.1** | **46.5 ± 2.6** | **65.0 ± 3.6** | **87.4 ± 4.1** |
| **E** | **40** | **0.5** | **95.6** | **36.9 ± 3.2** | **77.4 ± 6.7** | **89.8 ± 0.6** |
| F | 5 | 1.0 | 99.7 | 96.2 ± 2.9 | 25.4 ± 0.8 | NE |
| G | 10 | 1.0 | 99.5 | 83.5 ± 2.6 | 28.5 ± 0.9 | 87.5 ± 0.7 |
| H | 15 | 1.0 | 97.3 | 80.9 ± 10.7 | 41.5 ± 5.5 | 77.6 ± 0.2 |
| I | 20 | 1.0 | 99.3 | 53.8 ± 5.8 | 41.5 ± 4.5 | 84.4 ± 4.1 |
| J | 40 | 1.0 | 98.1 | 24.8 ± 4.7 | 23.9 ± 4.6 | 73.6 ± 8.1 |
| K | 5 | 3.0 | 99.4 | 59.6 ± 12.5 | 39.3 ± 8.4 | NE |
| **L** | **10** | **3.0** | **99.7** | **55.9 ± 10.6** | **50.7 ± 9.6** | **95.0 ± 1.6** |
| M | 15 | 3.0 | 99.4 | 39.8 ± 3.2 | 40.1 ± 3.2 | 86.3 ± 2.2 |
| N | 20 | 3.0 | 99.4 | 35.0 ± 5.3 | 35.3 ±5.3 | 66.2 ± 6.5 |
| O | 40 | 3.0 | 99.4 | 16.6 ± 5.6 | 66.6 ± 11.8 | 42.9 ± 2.9 |

[a] Immobilization yield (Ψ) = [(initial soluble activity –activity in the supernatant) / initial soluble activity] x 100. [b] Recovered activity was calculated as the percentage of hydrolytic activity (with pNPB as substrate), relative to the initial specific activity of the soluble enzyme. [c] Thermal stability is reported as residual activity after 2 h of incubation at 55 ºC. [d] IsoPro yield in *n*-heptane was carried out at 55 ºC and 180 rpm. NE: Not evaluated.

**Table S2.** Green and Sustainability Mass Metrics Summary.

| Metric | 5 mg CalB-CLEA (***n*-heptane**) | 1 g CalB ImmoPlus (**S/G system**) | 600 mg CalB-CLEA (**S/G system**) | 10 mg Novozym 435 (**Supercritical $CO_2$**) Varma et al., 2009[S1] | 1.85 g Novozym 435 (**Solvent-Free**) Kuperkar et al., 2014[S2] |
|---|---|---|---|---|---|
| **AE**[a] (%) | 87.9 | 87.9 | **87.9** | 87.9 | 87.9 |
| **Yield**[b] (%) | 97.8 | 98.2 | **91.3** | 95.0 | 92.5 |
| **Space Time Yield** ($g_{isoPro}$ $L^{-1}$ $h^{-1}$) | 6.2 | 11.8 | **8.2** | 7.1 | 48.4 |
| **RME**[c] (%) | 34.4 | 31.3 | **32.4** | 83.5 | 32.7 |
| **MP**[d] w/o $N_2$ (%) | 1.8 | 31.3 | **32.4** | 2.2 | 32.7 |
| **CE**[e] (%) | 36.0 | 32.6 | **34.0** | 95.0 | 34.2 |
| **E factor**[f] w/o $N_2$ | 55.7 | 3.8 | **3.2** | 45.5 | 2.2 |
| **E factor**[f] w/o $N_2$ and BSA | 55.6 | 3.8 | **2.1** | 45.5 | 2.2 |
| **Total Waste** w/o $N_2$ (g) | 7.1 | 2.4 | **1.7** | 67.7 | 26.8 |
| **Total Waste** w/o $N_2$ and BSA (g) | 7.1 | 2.4 | **1.1** | 67.7 | 26.8 |

[a] Atom Economy (%) = (mol wt of product/sum of mol of reactants)*100; [b] Yield (%) = (mol of product isoPro/mol of limiting reactant aciP)*100; [c] Reaction Mass Efficiency (%) = (Mass of product / Total mass of reactants)*100; [d] Mass Productivity (%) = (Mass product/Total mass including solvents)*100; [e] Carbon Economy (%) = (Carbon in product/Total carbon in reactants)*100; [f] E factor = Total mass of waste / Mass of final product. w/o = without.

## Supporting Figures

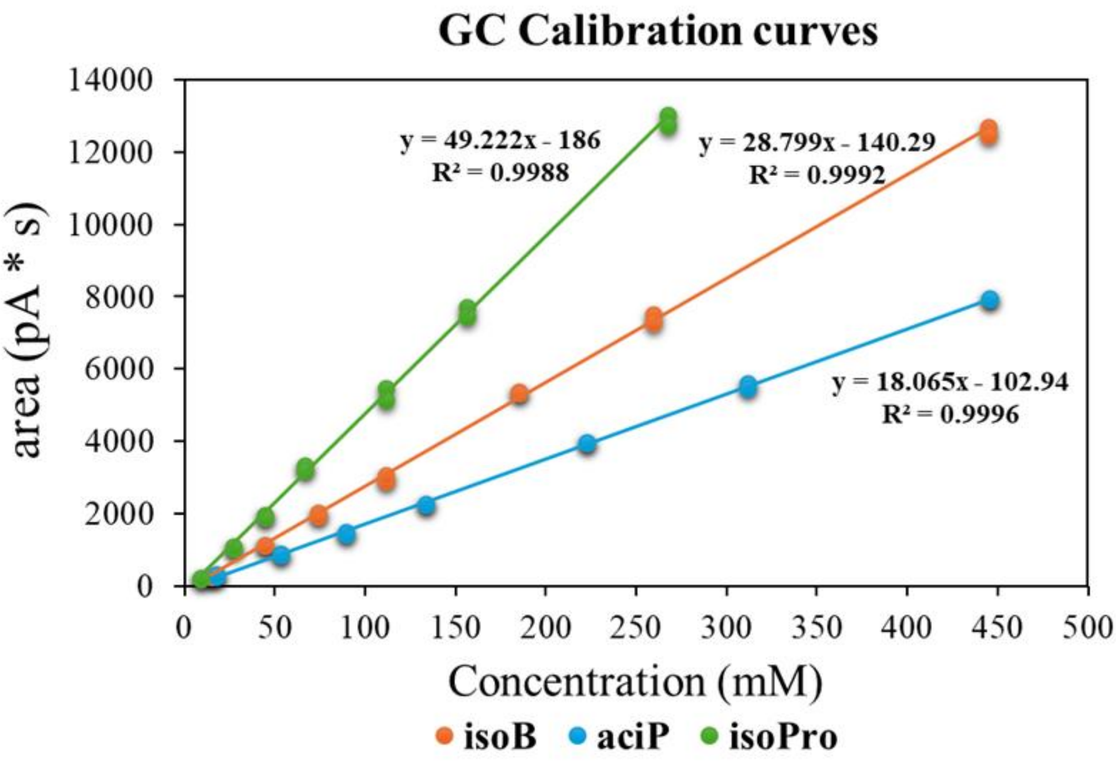


**Figure S1.** GC-FID calibration curves using an Agilent 7820 A and a DB-HEAVYWAX column (60 m × 0.25 mm i.d., 25 µm film thickness). Substrates (aciP and isoB) and product (isoPro).

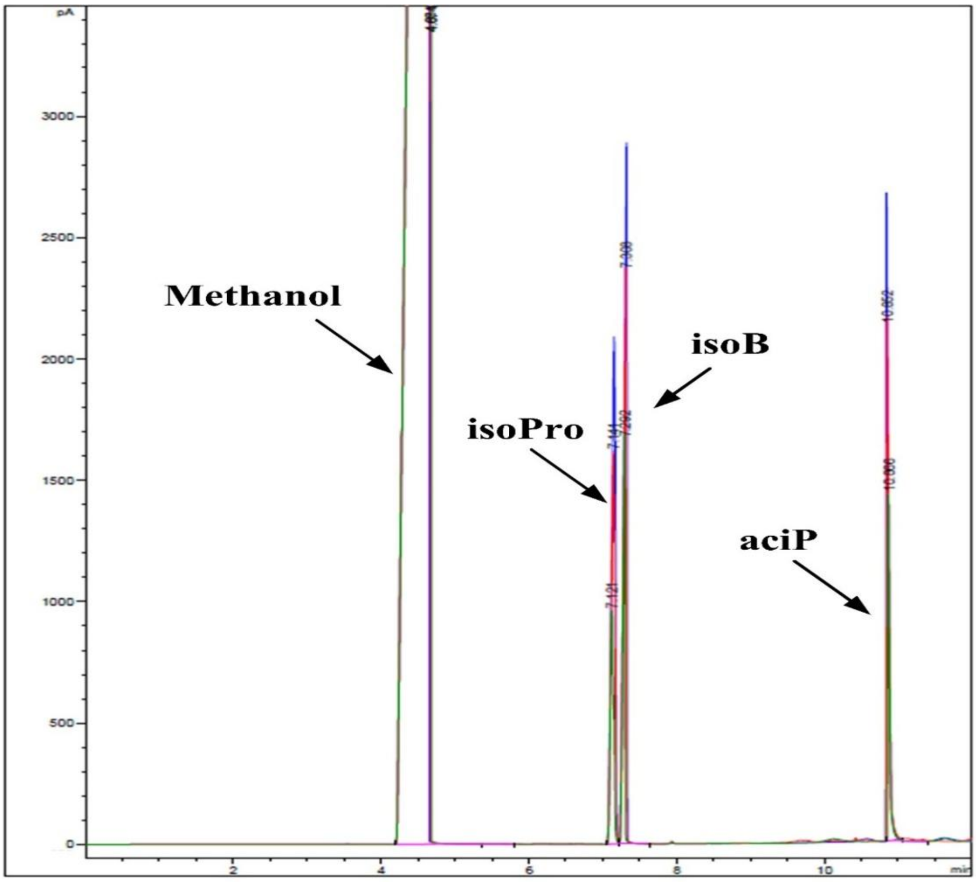


**Figure S2.** GC-FID analysis of commercial standards of isoPro, isoB, and aciP (Sigma-Aldrich). The standards were prepared at the following concentrations: isoPro (60, 110, and 150 mM), isoB (115, 200, and 280 mM), and aciP (140, 250, and 350 mM), represented by green, red, and blue traces, respectively. Retention times: 4.65 min for methanol, 7.13 min for isoPro, 7.29 min for isoB, and 10.85 min for aciP.

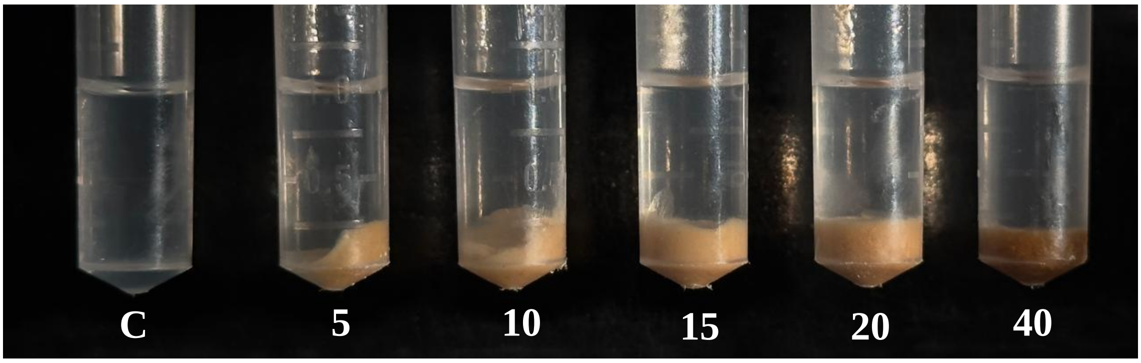


**Figure S3.** Effect BSA concentration (mg mL$^{-1}$) in CLEA body mass using 3% GA. (C: CalB without BSA).

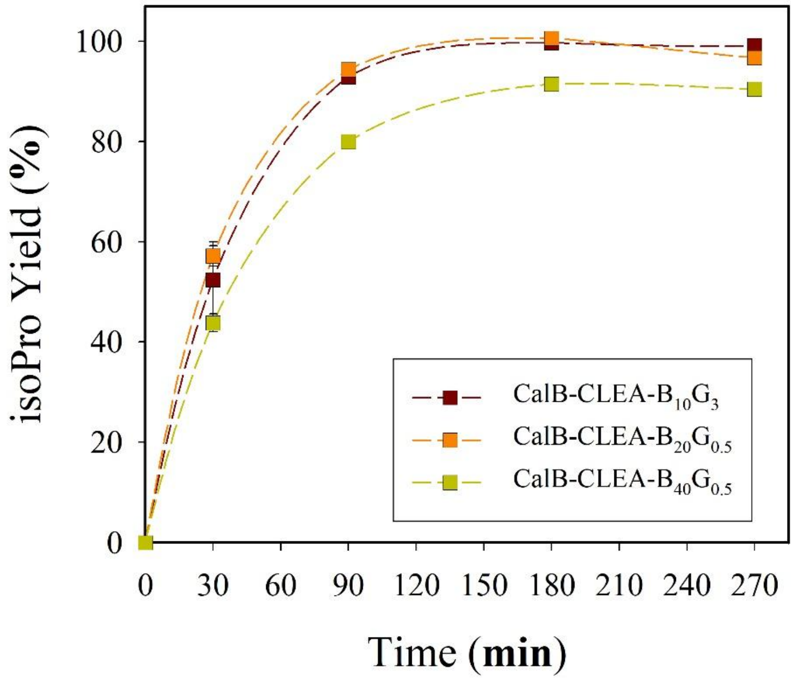


**Figure S4.** Comparison of isoPro synthesis by CalB-CLEA´s at $a_w$: 0.52 (400 mM isoB and 100 mM aciP in *n*-heptane) for 270 min at 55 °C and 180 rpm.

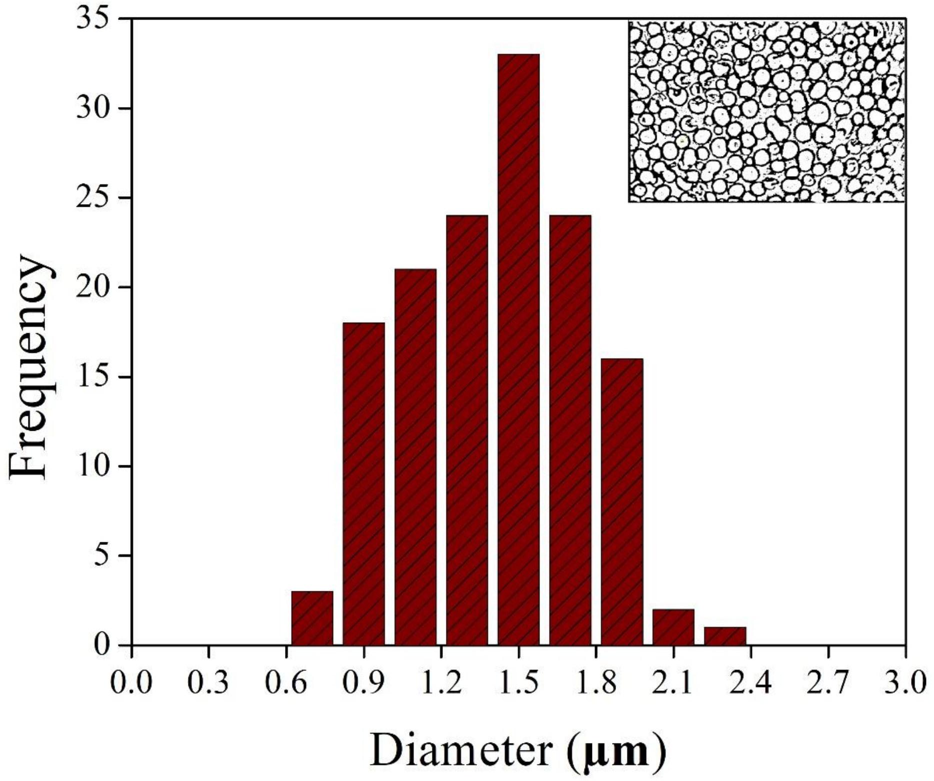


**Figure S5.** Particle size distribution of CalB-CLEA-$B_{10}G_3$ determined at a 1 µm scale and 3000 magnification SEM using ImageJ software.

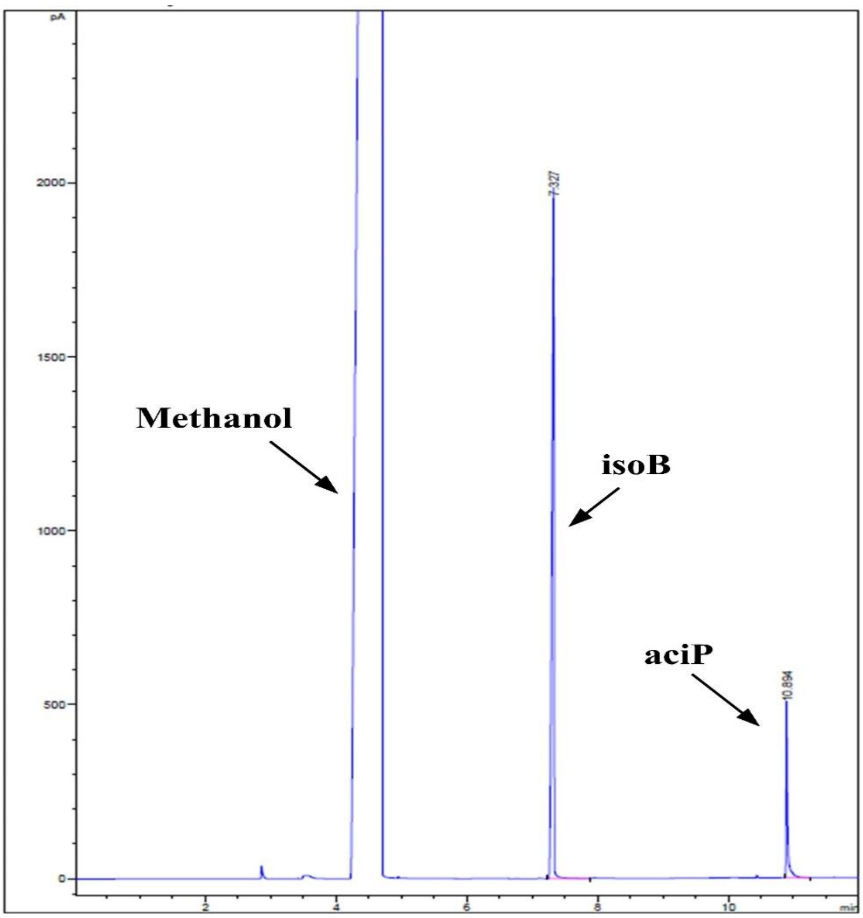


**Figure S6**. GC-FID analysis of the substrate feed introduced into the S/G bioreactor, based on a 4:1 molar ratio of isoB to aciP. The chromatogram was obtained after 2.5 minutes of outlet sampling in methanol, using an empty column.

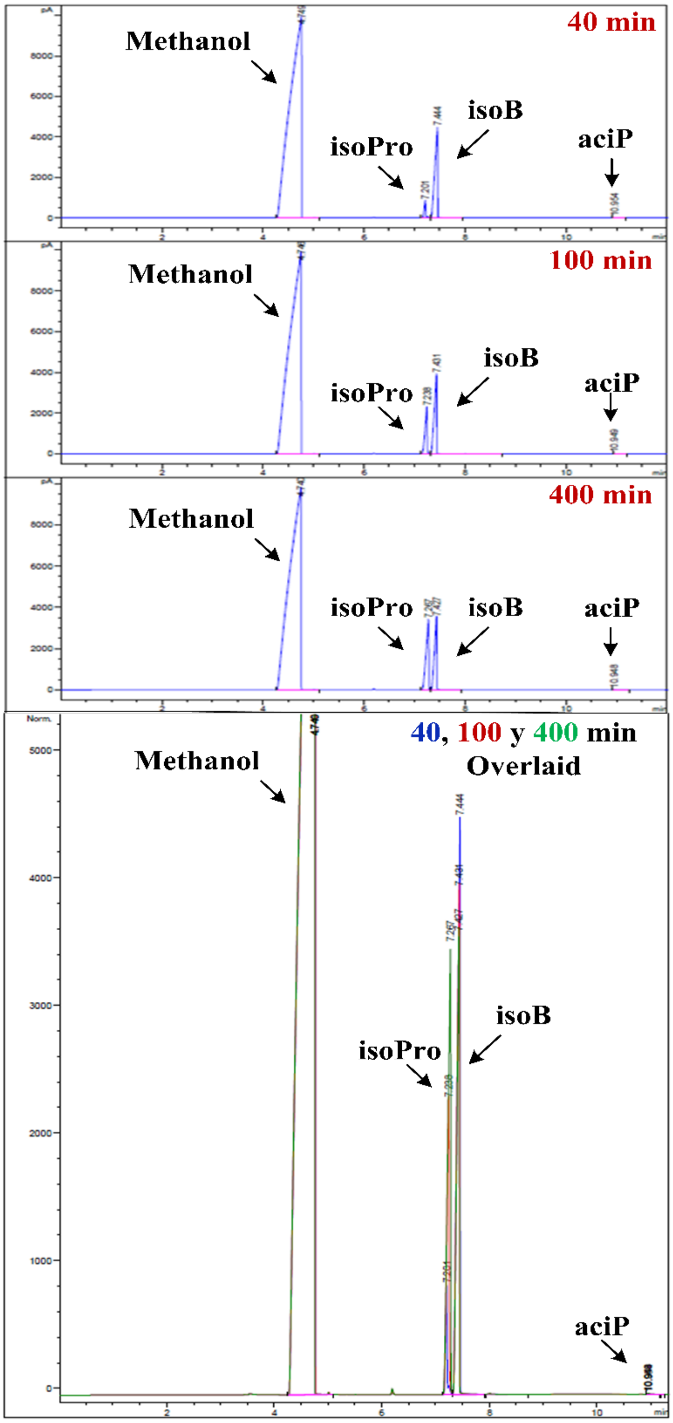


**Figure S7.** GC–FID monitoring of isoPro formation kinetics in a packed-bed CalB–CLEA reactor operated under the S/G system. Chromatograms at 40, 100, and 400 min (steady state) are shown individually and as overlaid plots in blue, red, and green, respectively. The aciP peak was scarcely detectable, consistent with high conversion efficiency and adsorption effects during the early reaction phase, as previously reported by Cruz-Martínez *et al.* (2024).[S3]

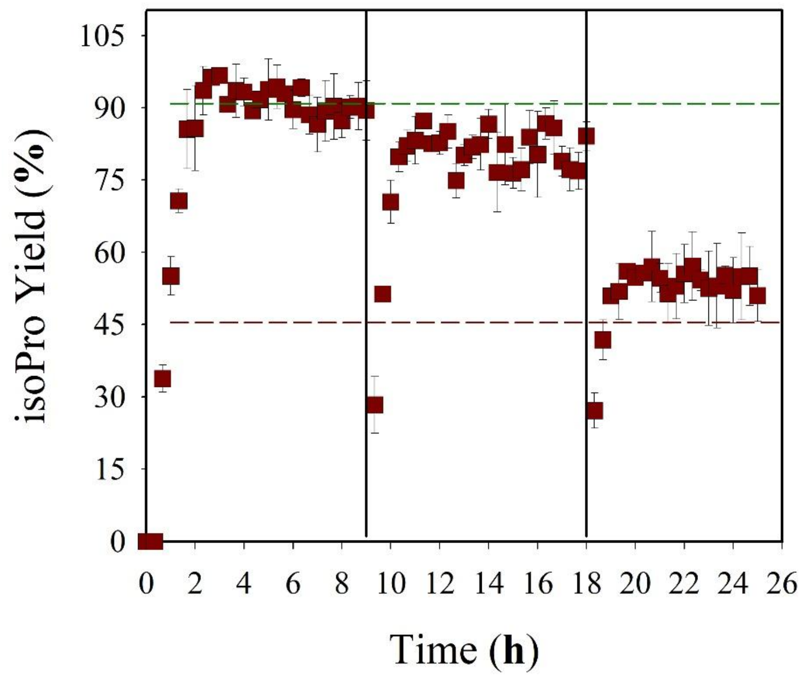


**Figure S8**. isoPro synthesis kinetics in the S/G system. The reactor contained 600 mg of CalB-CLEA-$B_{10}G_3$ at $a_w$: 0.52 and 55 °C. Data represent the mean of three independent replicates; error bars indicate standard deviation. Every 9 hours, the system was shut down, and the glass column containing the packed biocatalyst was removed from the flow setup and stored at 4 °C until the following day, without unpacking the catalyst.

## Supporting References

S1. Varma, M. N.; Madras, G. Effect of Chain Length of Alcohol on the Lipase-Catalyzed Esterification of Propionic Acid in Supercritical Carbon Dioxide. *Appl. Biochemistry and Biotechnology* **2010**, *160* (8), 2342–2354. DOI: 10.1007/s12010-009-8696-7

S2. Kuperkar, V. V.; Lade, V. G.; Prakash, A.; Rathod, V. K. Synthesis of Isobutyl Propionate Using Immobilized Lipase in a Solvent-Free System: Optimization and Kinetic Studies. *Journal of Molecular Catalysis B: Enzymatic* **2014**, *99*, 143–149. DOI: 10.1016/j.molcatb.2013.10.024.

S3. Cruz-Martínez, Y. A.; Castillo-Araiza, C. O.; Castillo-Rosales, E.; Huerta-Ochoa, S. Effect of Adsorption/Desorption of Substrates/Products during Isobutyl Propionate Synthesis over CalB Immo PlusTM in Solid/Gas Biocatalysis. *Biochemical Engineering Journal* **2024**, *208*, 109341. DOI: 10.1016/J.BEJ.2024.109341.
